# Effects of hip osteoarthritis on lower body joint kinematics during locomotion tasks: a systematic review and meta-analysis

**DOI:** 10.3389/fspor.2023.1197883

**Published:** 2023-11-17

**Authors:** Hannah Steingrebe, Sina Spancken, Stefan Sell, Thorsten Stein

**Affiliations:** ^1^ BioMotion Center, Institute of Sports and Sports Science, Karlsruhe Institute of Technology (KIT), Karlsruhe, Germany; ^2^ Sports Orthopedics, Institute of Sports and Sports Science, Karlsruhe Institute of Technology (KIT), Karlsruhe, Germany; ^3^ Joint Center Black Forest, Hospital Neuenbürg, Neuenbürg, Germany

**Keywords:** hip osteoarthritis, motion analysis, kinematics, gait, stair walking, turning, locomotion, clinical gait analysis

## Abstract

**Introduction:**

Motion analysis can be used to gain information needed for disease diagnosis as well as for the design and evaluation of intervention strategies in patients with hip osteoarthritis (HOA). Thereby, joint kinematics might be of great interest due to their discriminative capacity and accessibility, especially with regard to the growing usage of wearable sensors for motion analysis. So far, no comprehensive literature review on lower limb joint kinematics of patients with HOA exists. Thus, the aim of this systematic review and meta-analysis was to synthesise existing literature on lower body joint kinematics of persons with HOA compared to those of healthy controls during locomotion tasks.

**Methods:**

Three databases were searched for studies on pelvis, hip, knee and ankle kinematics in subjects with HOA compared to healthy controls during locomotion tasks. Standardised mean differences were calculated and pooled using a random-effects model. Where possible, subgroup analyses were conducted. Risk of bias was assessed with the Downs and Black checklist.

**Results and Discussion:**

A total of 47 reports from 35 individual studies were included in this review. Most studies analysed walking and only a few studies analysed stair walking or turning while walking. Most group differences were found in ipsi- and contralateral three-dimensional hip and sagittal knee angles with reduced ranges of motion in HOA subjects. Differences between subjects with mild to moderate and severe HOA were found, with larger effects in severe HOA subjects. Additionally, stair walking and turning while walking might be promising extensions in clinical gait analysis due to their elevated requirements for joint mobility. Large between-study heterogeneity was observed, and future studies have to clarify the effects of OA severity, laterality, age, gender, study design and movement execution on lower limb joint kinematics.

**Systematic Review Registration:**

PROSPERO (CRD42021238237).

## 1. Introduction

Hip osteoarthritis (HOA) is a common joint disease with high prevalence especially in the elderly (1). Due to pain and limited function HOA has a strong impact on the quality of life of the affected persons (2).

Data from instrumented movement analysis can be helpful for disease diagnosis as well as for the design and evaluation of patient-specific intervention strategies in HOA populations (3, 4). Depending on the data required, motion analysis can be obtained at different levels of complexity. While temporal-spatial gait characteristics are relatively easy to record (5) they might contain only limited informative value. Analysis of joint dynamics, first, requires simultaneous recording of ground reaction force data and, secondly, advanced methods of modelling (6). Due to the complexity and large time requirements, the applicability in the clinical setting might be limited (7). In 2018, Diamond et al. (8) reviewed the existing literature on external hip flexion and adduction moments in subjects with HOA. They found a reduction of both moments in patients with severe HOA but not in those with mild to moderate symptoms. Hence, adjustments of joint loading might take place at a later disease stage and thus might not be suitable for the early diagnosis of HOA. Similarly, Emmerzaal and colleagues (9) found lower or only slightly higher classification results between HOA and healthy subjects when joint dynamics combined with joint kinematics were used as input variables compared to kinematic variables only.

Therefore, analysis of joint kinematics might be the right balance between information acquisition and feasibility, especially in the clinical context. Thereby, wearable sensors’ increasing dissemination and popularity for the analysis of joint kinematics has also to be considered. With the availability of easy-to-use sensors and robust software tools for the estimation of joint angles, widespread usage of movement analysis in the clinical setting becomes feasible (10). In the past, several studies have shown kinematic changes at the hip joint in persons with varying degrees of HOA (11–13). Additionally, previous studies have shown that changes in gait patterns not only concern the hip joint itself but also the knee (14), ankle (15), pelvis (16) and upper body kinematics (17). Therefore, kinematics in general are of great interest for the characterisation of gait changes associated with HOA.

Gait, as one of the most fundamental movements of daily life, has often been used in the analysis of disease effects on movement kinematics, and previous reviews (4, 8, 18) focused on gait deviations caused by HOA. However, other locomotion tasks, such as stair ascent and descent might impose higher demands regarding the required range of motion (19), and thus their analysis might add essential insights into the movement restrictions and adaptations of people with HOA. A recently published article has shown that the most accurate classification between HOA subjects and healthy controls was done using joint angle data from stair walking trials compared to walking or stationary tasks such as lunges etc. (9).

Biomechanical gait analysis studies are often very time consuming (3, 4), and therefore mostly include only limited sample sizes (8). Additionally, subjects with HOA show heterogeneous disease characteristics such as varying degrees of functional and radiographic disease severity, uni- or bilateral involvement as well as primary or secondary HOA cause (8). An aggregation of multiple studies and collective evaluation of the results as well as the evaluation of specific subgroups might therefore add essential insights on the impact of HOA on movement biomechanics.

However, to the best of our knowledge, no comprehensive review exists regarding the kinematic changes observed during locomotion movements in the presence of HOA.

Therefore, the objective of the present systematic review was to summarise the current state of research on lower-limb joint kinematics during locomotion movements, such as gait or stair walking, in subjects diagnosed with HOA compared to healthy controls. Where possible a conjoint analysis of previous results in terms of a meta-analysis was performed, allowing special attention to be paid to the influence of HOA severity and uni- or bilateral involvement.

## 2. Materials and methods

This review was conducted according to the Preferred Reporting Items for Systematic Review and Meta-Analysis Statement (PRISMA) and registered in the International Prospective Register of Systematic Reviews (PROSPERO, no. CRD42021238237).

### 2.1. Information sources, search strategy and screening process

Eligible reports were searched in three electronic databases [PubMed (MEDLINE, PubMed Central, and additional PubMed records), Web of Science and Scopus] on August 2nd 2022.

The title, abstract and keywords were screened for disease description (Coxarthr*, “degenerative joint” AND hip, hip AND osteoarthr*), the outcome parameter (kinematic*, angle*, “range of motion”, mobility, pattern, goniometric*, biomechanic*) and the movement task (gait, walk*, locomotion, ambulat*, stair*, movement). Titles including *fracture*, *perthes*, *amputee*, rheumat*, *arthroscopy or arthroplasty* were excluded. The detailed search term for each database can be found in the Supplementary Material.

All records from the databases were imported into a reference manager (Citavi 6, Swiss Academic Software GmbH, Switzerland) and duplicates were removed. The reference lists of included articles were screened manually for additional eligible studies.

The literature search, title and abstract screening as well as full text analysis for eligibility were performed individually by two researchers (HS & SSp). Discrepancies were resolved by discussion between the two authors, and if no consensus was reached a third researcher (TS) was consulted.

### 2.2. Eligibility criteria

Original research studies written in English, evaluating pelvis or lower extremity (hip, knee, ankle) joint kinematics during locomotion movements (e.g., level walking, running or stair walking) in a cohort of subjects with HOA were eligible for this review. No restrictions were made regarding the OA severity, unilateral or bilateral involvement or whether the HOA was primary or secondary. Data from the HOA subjects had to be compared to a healthy control group (CON). Accepted outcome parameters were a quantitative description of lower body joint angle parameters (mean/median with measure of dispersion) and/or the presence of *p*-values from the comparison of lower body joint angle parameters. Studies with an intervention were included using the pre-intervention data if applicable. A detailed description of the eligibility criteria can be found in Table 1.

**Table 1:** Description of the inclusion and exclusion criteria following a modified PECO scheme.

	Inclusion criteria	Exclusion criteria
Population	Humans with hip osteoarthritis (uni- & bilateral)	Subjects with other diseases (e.g. hip dysplasia, knee OA) or endoprosthesis
Exposure	Analysis of a locomotion tasks (e.g. gait, stair walking)	Stationary movements (e.g. sit to stand, one-leg stand), use of walking aids or handrails
Comparator	Healthy control group	No control group or contralateral limb as control
Outcome	Report of kinematic data on lower body joint angles (hip, knee, ankle) or pelvis movement	Sole report of temporal-spatial-parameters or other kinematic data (e.g. toe-out angles), or analyses of kinematic coordination (whole body analysis, coupling angles, asymmetry measures)
Language	English language	Other languages
Format	Original full text paper	Reviews, conferences proceedings, case studies etc.

### 2.3. Data collection process

One researcher (HS) extracted the data from the retrieved reports using a predefined spreadsheet including the following sections: study design, number of HOA and CON subjects, participant characteristics (i.e., age, gender, weight, height, body mass index, radiographic disease severity, functional disease severity, uni-/bilateral involvement, primary/secondary OA), analysed movements (e.g., walking, stair climbing) and testing conditions (treadmill or overground, number of stairs, prescribed or self-selected movement velocity), measurement system and assessed joints (e.g., hip sagittal plane). Extracted data were randomly cross-checked by a second researcher (SSp).

The extracted data were analysed for indications of multiple reports from the same study. In case of doubt, report authors were contacted for clarification. Extracted data of the kinematic variables were summarised in tables and grouped according to planes of motion and joint location.

Disease severity was categorised as mild, moderate or severe based on the information of the reports. End-stage HOA as well as subjects scheduled for total hip replacement were classified as severe.

### 2.4. Synthesis methods

Peak angles were converted if necessary to obtain a unified definition of the angle direction. If both limbs were reported for the CON subjects, data from the right limb were used. One study (20) reported joint angles of two different examiners to calculate inter-rater reliability. For the meta-analysis, values of examiner 1 were extracted.

Confidence intervals for group means were transformed to standard deviations following the Cochrane handbook (21).

If mean and standard deviation were available, standardised mean differences (SMD) and 95% confidence intervals (95% CI) were calculated for all variables by dividing the difference between groups by the pooled standard deviation (effect size Cohen’s d). Where possible (≥2 studies) pooled effect sizes were calculated using a random-effects model with a restricted maximum likelihood estimator and Knapp-Hartung adjustment. The random-effects model was applied to account for variability in the composition of the subject groups (e.g., HOA severity, gender etc.) and the movement execution (e.g., movement speed). SMDs and pooled SMDs ≥0.2 were interpreted as a small effect, ≥0.5 as a moderate effect and ≥0.8 as a large effect (22). Statistical heterogeneity was evaluated from pooled data using the *I*^2^ statistic, with a value of 25% considered low, 50% considered moderate and 75% considered a high level of heterogeneity (23). Additionally, prediction intervals were reported (24, 25). Subgroup analyses for HOA severity and laterality (uni-/bilateral) were conducted if data from ≥2 studies for ≥2 clearly distinguishable subgroups were available. If multiple HOA subgroups from the same study were included, the sample size of the CON group was split equally to all HOA subgroups. For all analyses, the significance level was set *a priori* to *ɑ* < 0.05. The meta-analysis (including calculation of *I*^2^ and prediction intervals) was conducted in R (version 4.2.2) using the meta package.

For several studies, multiple effect sizes were available due to the presence of multiple reports including equal or overlapping subject samples, analyses of subgroups (e.g., men and women) or analyses of different movement conditions (e.g., walking speeds). In those cases, where data of independent subgroups was presented (e.g., OA severity, gender), we recreated the summary data (weighted mean & combined standard deviation) prior to effect size calculation (26). If the presented data were of dependent subgroups (e.g., multiple reports for one study, multiple walking speeds analysed), one study was selected and included in the meta-analysis based on the following criteria: (I) largest overall sample size, (II) movement condition most similar to other studies (27), (III) self-selected gait speed.

Data from studies that could not be synthesised in a meta-analysis were synthesised qualitatively.

### 2.5. Risk of bias & quality of reporting

Risk of bias and quality of reporting of all included studies were assessed individually by two researchers (HS & SSp) using the checklist created by Downs and Black (28). Of the 27 items, 11 concerning interventions were removed (items 4, 8. 9, 13, 14, 15, 17, 19, 23, 24, 26) as previously done (8). This resulted in a maximum score of 17, with higher scores representing a lower risk of bias. For question 5, age, weight or BMI and gender were defined as principal confounders. Question 27 was answered yes if an *a priori* or *post-hoc* power analysis was reported. Disagreements in initial ratings were discussed by HS and SSp to reach consensus. If no consensus was reached a third reviewer (TS) was consulted.

## 3. Results

**Figure 1:**
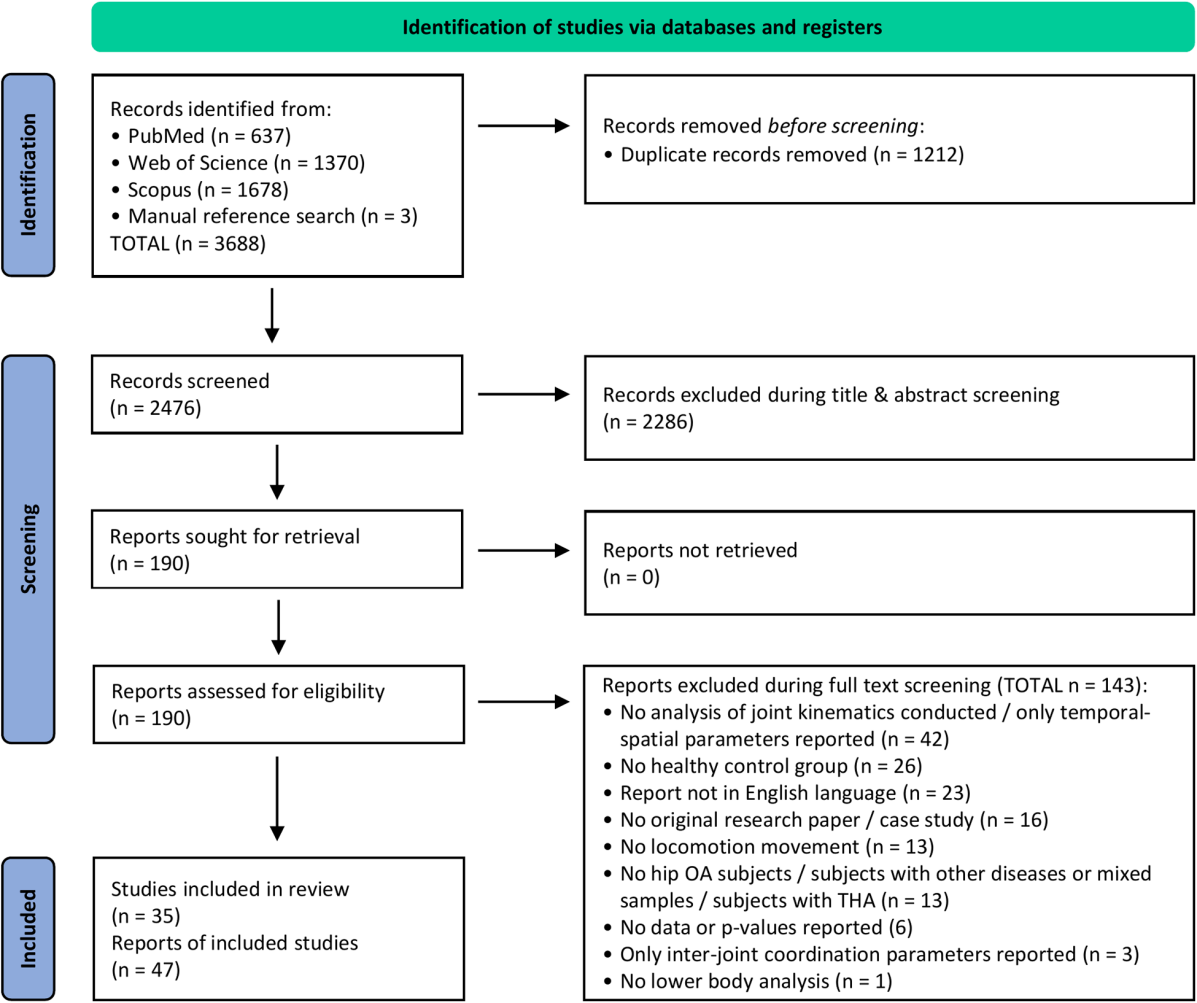
PRISMA flow diagram (29).

The process of study identification and screening is displayed in Figure 1. In total, 47 reports representing 35 independent studies met the inclusion criteria and are reviewed below. Details of all included reports are listed in Tables 2, 3. In total, the studies included 949 subjects with HOA and 886 CON subjects. For 3.8% of the HOA subjects and 2.1% of the CON subjects, gender was not reported. Of the remaining subjects, 56.7% of HOA subjects and 62.7% of CON subjects were female.

**Table 2:** Detailed population characteristics of the included studies. All data are presented as mean ± standard deviation.

Study	Report	Population characteristics						
		Group	*n* (♀)	age [years]	height [cm]	weight [kg]	BMI [kg/m^2^]	OA characteristics
								(severity, laterality, cause)
A	Aminian et al. (30)	CON	9 (–)	63 ± 4	161 ± 10	63 ± 9		Severe; unilateral
		HOA	11 (–)	60 ± 9	167 ± 5	74 ± 8		
B	Ardestani & Wimmer (31)	CON	23 (13)	58.2 ± 9.7			27.98 ± 3.9	Mild, Moderate, Severe (Subgroups); unilateral
		HOA	45 (20)	59 ± 11				
C	Baker et al. (32)	CON	20 (–)	63 ± 6	172 ± 9	76.0 ± 18.4	25.6 ± 5.0	Moderate, Severe (Subgroups); unilateral
		HOA	36 (–)	61.2 ± 3.8	170.4 ± 2.7	84.2 ± 4.1	28.9 ± 2.0	
	Rutherford et al. (14)	CON	20 (10)	63 ± 6	172 ± 9	76 ± 18.4	25.6 ± 5.1	Moderate; unilateral
		HOA	20 (5)	59 ± 8	170 ± 8	83 ± 16.8	28.7 ± 4.3	
	Rutherford et al. (33)	CON	20 (–)	62 ± 6			25.6 ± 5	Moderate, Severe (Subgroups); unilateral
		HOA	37 (–)	60.8 ± 3.4			29.3 ± 2.2	
D	Bejek et al. (34)	CON	20 (12)	68.8 ± 9.1	169 ± 19	73.3 ± 11.4		Severe; unilateral
		HOA	20 (12)	69.7 ± 8.9	172 ± 11	70.1 ± 9.1		
E	Benedetti et al. (35)	CON	10 (4)	59				Severe; -
		HOA	8 (3)	48.7	173	78.5		
F	Bolink et al. (36)	CON	20 (11)	61.0 ± 6.1	173 ± 8.4	77.2 ± 12.7	25.8 ± 3.0	Severe; unilateral
		HOA	20 (10)	63.4 ± 8.5	172 ± 9.7	81.1 ± 17.8	27.2 ± 4.9	
G	Brand & Crowninshield (37)	CON	15 (7)	71				
	(CON data: Crowninshield et al.) (38)	HOA	8 (–)					
H	Constantinou et al. (39)	CON	26 (18)	59.3 ± 7.6	169 ± 8	70.5 ± 9.3	24.8 ± 3.0	Mild/Moderate; mixed (11 bi, 16 uni)
		HOA	27 (18)	63.2 ± 7.6	166 ± 9	77.6 ± 14.2	28.0 ± 4.1	
	Diamond et al. (13)	CON	23 (17)	60 ± 8	167 ± 8	69.9 ± 9.5	25.1 ± 3.1	Mild/Moderate; mixed (9 bi, 9 uni)
		HOA	18 (13)	65 ± 7	166 ± 10	76.2 ± 14.1	27.6 ± 4.8	
I	Eitzen et al. (11)	CON	22 (13)	58.5 ± 8.8	171.7 ± 10.8	70.8 ± 15.1	23.8 ± 3.5	Mild/Moderate; mixed (10 bi, 38 uni)
		HOA	48 (29)	59.1 ± 9.5	172.3 ± 8.4	73.2 ± 12.2	24.6 ± 3.3	
J	Foucher (40)	CON	159 (104)	55.7 ± 2.9			26.7 ± 2.3	Mixed; -
		HOA	150 (86)	62.3 ± 3.4			28.3 ± 2.3	
K	Foucher et al. (41)	CON	25 (11)	57.6 ± 7.7	173 ± 9	79.2 ± 17.8		Severe; unilateral
		HOA	28 (10)	63.6 ± 7.1	172 ± 11	86.7 ± 15.8		
L	Foucher & Wimmer (42)	CON	25 (–)	54 ± 6			28 ± 6	Severe; unilateral; primary
		HOA	26 (–)	59 ± 9			27 ± 3	
	Foucher et al. (43)	CON	25 (13)	54 ± 6	171 ± 6.9	81.6 ± 13.3	28 ± 6	Severe; unilateral; primary
		HOA	26 (11)	60 ± 4.2	175.5 ± 6.3	83.9 ± 5.9	27 ± 3	
M	Hall et al. (44)	CON	15 (11)	64.0 ± 8.7	163 ± 11	72.6 ± 11.6	23.8 ± 3.6	Mixed; mixed (9 bi, 6 uni)
		HOA	15 (8)	63.1 ± 6.7	168 ± 11	76.0 ± 11.8	28.1 ± 4.0	
N	Hara et al. (45)	CON	6 (0)	33	173	67		Severe; -
	(CON data: Hara et al. (46)	HOA	14 (12)	65 ± 7	154 ± 8	54 ± 9	23 ± 4	
O	Hurwitz et al. (47)	CON	19 (7)	61 ± 8	173 ± 10	72.4 ± 15.4		Severe; unilateral
		HOA	19 (7)	60 ± 8	170 ± 10	79.2 ± 11.2		
P	Ismailidis et al. (48)	CON	48 (30)	66.6 ± 7.2	169 ± 9	71.6 ± 12.5	25.1 ± 4.0	Severe; unilateral
		HOA	24 (10)	66.1 ± 10.3	171 ± 8	80.8 ± 13.3	27.5 ± 3.2	
	Ismailidis et al. (49)	CON	45 (29)	66.6 ± 7.4	169 ± 8	71.0 ± 11.9	25.0 ± 4.1	Severe; unilateral
		HOA	22 (10)	66.3 ± 10.2	171 ± 8	79.7 ± 13.2	27.3 ± 3.3	
	Nüesch et al. (50)	CON	54 (23)	66.4 ± 7.9	170 ± 9	73.1 ± 13.5	25.3 ± 4.0	Severe; unilateral
		HOA	30 (18)	64.9 ± 11.6	172 ± 8	80.6 ± 12.8	27.1 ± 3.1	
Q	Kataoka et al. (51)	CON	15 (15)	61.2 ± 6.3	155.8 ± 3.7	53.5 ± 7.3		Moderate/Severe; -
		HOA	15 (15)	60.4 ± 9.6	152.8 ± 2.9	57.1 ± 11.4		
R	Kubota et al. (52)	CON	12 (12)	64.3 ± 2.8	148.1 ± 5.1	52.5 ± 7.8	23.9 ± 2.8	Moderate/Severe; bilateral
		HOA	12 (12)	59.4 ± 11.1	150.6 ± 6.1	56.7 ± 7.1	24.9 ± 3.4	
S	Kumar et al. (12)	CON	30 (16)	48.2 ± 11.4			23.3 ± 3.3	Mild/Moderate; mixed
		HOA	36 (12)	54.5 ± 8.9			24.5 ± 3.0	
T	Leigh et al. (15)	CON	22 (13)	53.7 ± 8.3	168.8 ± 9.5	76.5 ± 9.4	26.8 ± 1.5	Mild/Moderate; mixed (6 bi, 16 uni)
		HOA	22 (12)	55.9 ± 7.5	170.2 ± 8.0	76.8 ± 15.4	26.5 ± 4.6	
U	Meyer et al. (53)	CON	17 (8)	52.7 ± 4.9	171 ± 10		24.1 ± 2.7	Severe; unilateral
		HOA	20 (5)	49.7 ± 9.5	173 ± 10		25.5 ± 3.2	
	Wesseling et al. (54)	CON	18 (9)	53.0 ± 5.0	171 ± 10	69.3 ± 12.5	23.7 ± 3.1	Severe; unilateral
		HOA	14 (5)	47.3 ± 11.8	173 ± 9	76.3 ± 16.6	25.2 ± 3.7	
V	Ornetti et al. (55)	CON	9 (7)	60.3 ± 7				Mixed; unilateral; primary
		HOA	11 (8)	60.5 ± 7			25.7 ± 6	
W	Popovic et al. (56)	CON	30 (16)	44.7 ± 13.5			23.7 ± 2.4	Mild/Moderate; -
		HOA	42 (23)	49.6 ± 15.2			24.5 ± 3.3	
X	Porta et al. (57)	CON	11 (5)	67.8 ± 5.4	165.9 ± 7.6	75.9 ± 10.1		Severe; -; primary
		HOA	11 (5)	68.3 ± 5.8	162.0 ± 5.4	75.4 ± 16.9		
Y	Reininga et al. (17)	CON	30 (22)	66 ± 6	170 ± 9	69 ± 12		Severe; -
		HOA	60 (45)	59.7 ± 3.3	170.8 ± 2.7	77.7 ± 3.5		
Z	Schmidt et al. (58)	CON	18 (7)	60.4 ± 8.9	173.1 ± 8.9	71.5 ± 13.7	23.7 ± 3.0	Severe; unilateral
		HOA	18 (7)	64.4 ± 7.4	170.9 ± 7.7	80.7 ± 14.4	27.5 ± 3.4	
	Stief et al. (59)	CON	15 (6)	61.5 ± 8.0	174 ± 9	71.7 ± 14.7	23.5 ± 2.9	Severe; unilateral
		HOA	15 (6)	65.9 ± 8.6	172 ± 8	79.7 ± 9.9	27.0 ± 2.3	
AA	Schmitt et al. (60)	CON	15 (7)	49.2 ± 7.1	166 ± 18	67.4 ± 11.6		Severe; unilateral
		HOA	30 (15)	54.8 ± 6.7	172 ± 10	83.2 ± 20.6		
AB	Steingrebe et al. (61)	CON	21 (10)	63.1 ± 9.2	171.1 ± 8.8	74.4 ± 12.7	25.2 ± 2.7	Mild/Moderate; unilateral
		HOA	21 (10)	64.0 ± 9.6	171.2 ± 6.7	71.3 ± 11.9	24.2 ± 2.9	
AC	Tanaka (62)	CON	56 (56)					-; unilateral, bilateral (subgroups)
		HOA	24 (24)					
AD	Tateuchi et al. (63)	CON	13 (13)	62.6 ± 4.4	152.7 ± 4.9	50.6 ± 5.3		Severe; -
		HOA	14 (14)	59.3 ± 5.3	153.3 ± 5.5	53.3 ± 9.1		
AE	Thurston (64)	CON	10 (-)	63.4 ± 8.1				Severe; unilateral
		HOA	20 (0)	65.1 ± 7.8				
Z & AF	van Drongelen et al. (65)	CON	26 (16)	63.3 ± 7.9	168 ± 10	69.3 ± 12.8	24.6 ± 3.1	Severe; unilateral, bilateral (subgroups)
		HOA	52 (32)	64.3 ± 3.1	169 ± 3.1	77.9 ± 4.0	27.1 ± 2.1	
	van Drongelen et al. (66)	CON	46 (25)	64.2 ± 7.0	169 ± 10	69.0 ± 12.6	24.2 ± 2.8	Severe; unilateral
		HOA	51 (21)	60.6 ± 9.9	173 ± 7	80.3 ± 11.5	26.7 ± 2.9	
AF	van Drongelen et al. (67)	CON	18 (7)	60.4 ± 8.0	173 ± 9	72.0 ± 13.9	23.9 ± 3.2	Severe; unilateral
		HOA	17 (8)	60.5 ± 9.9	172 ± 9	83.3 ± 15.9	28.1 ± 4.9	
	van Drongelen et al. (68)	CON	15 (6)	61.5 ± 8.9	174 ± 9	71.7 ± 14.7	23.5 ± 2.9	Severe; unilateral
		HOA	22 (13)	62.3 ± 10.2	171 ± 10	82.4 ± 16.7	28.2 ± 4.9	
AG	Watanabe et al. (69)	CON	54 (54)	29.4				Mild/Moderate; unilateral
		HOA	30 (30)	31.2				
	Watanabe et al. (70)	CON	54 (54)	29.4 ± 4.6				Mild/Moderate; unilateral
		HOA	30 (30)	31.2 ± 5.6				
AH	Watelain et al. (16)	CON	17 (9)	63.6 ± 5.2	169 ± 6	71.1 ± 14.0		Severe; unilateral
		HOA	17 (9)	58.9 ± 7.1	167 ± 12	76.6 ± 14.7		
AI	Zügner et al. (20)	CON	20 (10)	45.4 ± 4.4			24.3 ± 1.4	Severe; unilateral
		HOA	20 (10)	58.5 ± 5.2			28.0 ± 2.5	

CON, control group; HOA, hip osteoarthritis group.

**Table 3:** Details on osteoarthritis (OA) assessment method and study design of included studies.

		OA assessment		Study design		
Study	Report	Radiographic	Functional	Movement	Measurement system	Analyzed joints
A	Aminian et al. (30)		HHS	Gait OG	IMU	Knee (I & C)
B	Ardestani & Wimmer (31)	KL		Gait OG	Optoelectronic	Hip (I), Knee (I), Ankle (I)
C	Baker et al. (32)	KL	HOOS	Gait TM	Optoelectronic	Hip (I)
	Rutherford et al. (14)		HOOS, WOMAC	Gait TM	Optoelectronic	Knee (I & C)
	Rutherford et al. (33)			Gait TM	Optoelectronic	Hip (I)
D	Bejek et al. (34)	KL	HHS	Gait TM	Ultrasound	Hip (I & C), Knee (I & C), Pelvis
E	Benedetti et al. (35)			Gait OG	Optoelectronic	Hip (I), Pelvis
F	Bolink et al. (36)	KL		Gait OG	IMU	Pelvis
G	Brand & Crowninshield (37) (CON data: Crowninshield et al.) (38)			Gait OG	Biplanar photography	Hip (I)
H	Constantinou et al. (39)	KL	HHS	Gait OG	Optoelectronic	Hip (I), Pelvis
	Diamond et al. (13)	KL	HHS	Gait OG	Optoelectronic	Hip (I)
I	Eitzen et al. (11)			Gait OG	Optoelectronic	Hip (I), Knee (I), Ankle (I)
J	Foucher (40)	KL		Gait OG	Optoelectronic	Hip (I)
K	Foucher et al. (41)		HHS	Gait OG	Optoelectronic	Hip (I)
L	Foucher & Wimmer (42)			Gait OG	Optoelectronic	Hip (C), Knee (C)
	Foucher et al. (43)			Gait OG	Optoelectronic	Hip (I)
M	Hall et al. (44)	KL	HOOS	Stairs (A & D)	Optoelectronic	Hip (I), Pelvis
N	Hara et al. (45)	KL		Gait TM	Continuous radiographic imaging	Hip (I), Pelvis
	(CON data: Hara et al. (46)					
O	Hurwitz et al. (47)		HHS	Gait OG	Optoelectronic	Hip (I & C)
P	Ismailidis et al. (48)	KL	HOOS	Gait OG	IMU	Hip (I), Knee (I), Ankle (I)
	Ismailidis et al. (49)	KL	HOOS	Gait OG	IMU	Hip (I & C), Knee (I & C), Ankle (I & C)
	Nüesch et al. (50)	KL	HOOS	Gait OG	IMU	Hip (I & C), Knee (I & C), Ankle (I & C)
Q	Kataoka et al. (51)	KL	HHS	Gait TM	IMU	Hip (I), Knee (I), Ankle (I)
R	Kubota et al. (52)	KL	JOA HS	Gait OG	Optoelectronic	Hip (I), Ankle (I), Pelvis
S	Kumar et al. (12)	KL	HOOS	Gait OG	Optoelectronic	Hip (I)
T	Leigh et al. (15)	KL	WOMAC	Gait TM	Optoelectronic	Hip (I), Knee (I), Ankle (I), Pelvis
U	Meyer et al. (53)	Tönnis		Gait OG	Optoelectronic	Hip (I)
	Wesseling et al. (54)			Gait OG	Optoelectronic	Hip (I & C), Knee (I & C), Ankle (I & C)
V	Ornetti et al. (55)	KL		Gait OG	Optoelectronic	Hip (I & C), Knee (I & C), Ankle (I & C)
W	Popovic et al. (56)	KL	HOOS	Stairs (A & D)	Optoelectronic	Hip (I), Knee (I), Ankle (I)
X	Porta et al. (57)	KL		Gait OG	Optoelectronic	Hip (I & C), Knee (I & C), Ankle (I & C)
Y	Reininga et al. (17)			Gait OG	IMU	Pelvis
Z	Schmidt et al. (58)	KL	HHS	Gait OG	Optoelectronic	Knee (I & C)
	Stief et al. (59)	KL	HHS	Gait OG	Optoelectronic	Hip (I & C), Knee (I & C), Pelvis
AA	Schmitt et al. (60)			Gait OG	Optoelectronic	Hip (I), Knee (I), Ankle (I)
AB	Steingrebe et al. (61)	KL	HHS, HOOS	Gait OG	Optoelectronic	Hip (I), Pelvis
AC	Tanaka (62)			Gait OG	Optoelectronic	Hip (I & C)
AD	Tateuchi et al. (63)		HHS	Gait OG, Turning 45°	Optoelectronic	Hip (I), Knee (I), Ankle (I)
AE	Thurston (64)			Gait OG	Video camera	Pelvis
Z & AF	van Drongelen et al. (65)	KL	HOOS, HHS	Gait OG	Optoelectronic	Hip (I & C), Knee (I & C), Ankle (I & C), Pelvis
	van Drongelen et al. (66)		HOOS, HHS	Gait OG	Optoelectronic	Hip (I), Knee (I), Pelvis
AF	van Drongelen et al. (67)	KL		Gait OG	Optoelectronic	Hip (I & C), Knee (I & C), Pelvis
	van Drongelen et al. (68)			Gait OG	Optoelectronic	Hip (I & C), Knee (I & C)
AG	Watanabe et al. (69)			Gait OG	Optoelectronic	Pelvis
	Watanabe et al. (70)			Gait OG	Optoelectronic	Pelvis
AH	Watelain et al. (16)	KL	Lequesne Index	Gait OG	Optoelectronic	Hip (I), Pelvis
AI	Zügner et al. (20)	Ahlbäck		Gait OG	Optoelectronic	Hip (I)

KL, Kellgren-Lawrence-Score; HHS, Harris Hip Score; HOOS, Hip Osteoarthritis Outcome Score; WOMAC, Western Ontario and McMaster Universities Osteoarthritis Index; JOA HS, Japanese Orthopaedic Association hip score; OG, overground; TM, treadmill; IMU, inertial measurement unit; I, ipsilateral; C, contralateral.

Only 1 study included subjects with mild HOA, 7 with mild to moderate HOA, 2 with moderate HOA, 2 studies subjects with moderate to severe HOA and 21 with severe HOA. In 3 studies subjects with varying degrees of HOA were included and 2 studies gave no details on HOA severity.

In 20 studies subjects with unilateral HOA were included and 2 studies included those with bilateral HOA. Mixed samples were included in 5 studies and 9 studies did not give information on limb involvement. In 3 studies subjects were diagnosed with primary HOA. For all other studies, no information regarding primary or secondary OA was available.

Gait movement was analysed in 33 studies, of which 28 analysed overground walking, while 5 studies analysed walking on a treadmill. Two studies analysed stair ascent and descent and 1 study analysed turning while walking.

Hip kinematics were analysed in 31 studies, knee kinematics in 18 studies, ankle kinematics in 13 studies and pelvis kinematics in 16 studies. Thereof, 10 studies (hip and knee), 5 studies (ankle) and 2 studies (pelvis) also analysed contralateral joint kinematics.

Data of 29 studies (27 studies on gait, 2 studies on stair walking) were included in the meta-analysis. For the sake of brevity, only forest plots of analyses with significant results are included in the text, and forest plots for all other analyses can be found in the Supplementary Material.

### 3.1. Risk of bias & quality of reporting

The results of the risk of bias assessment are presented in Table 4. The mean score was 10.5 (±2.3) with the minimum being 2 and the maximum being 15 (maximum achievable = 17). We did not exclude any of the studies on the basis of their total score.

**Table 4:** Results of the risk of bias assessment using the downs & black checklist (28).

Study	Report	Q1	Q2	Q3	Q5	Q6	Q7	Q10	Q11	Q12	Q16	Q18	Q20	Q21	Q22	Q25	Q27	Total	Total [%]
A	Aminian et al. (30)	1	1	0	1	1	1	0	0^a^	0^a^	1	0^a^	1	0^a^	0^a^	0	0	7	41
B	Ardestani & Wimmer (31)	1	1	1	2	1	1	1	0	0^a^	1	0^a^	1	0^a^	1	1	0	12	71
C	Baker et al. (32)	1	1	1	1	1	1	1	0^a^	0^a^	1	1	1	0	0^a^	1	0	11	65
	Rutherford et al. (14)	1	1	1	2	1	1	1	0^a^	0^a^	1	1	1	0	0^a^	1	0	12	71
	Rutherford et al. (33)	1	1	1	1	1	1	1	0^a^	0^a^	1	1	1	0	0^a^	1	0	11	65
D	Bejek et al. (34)	1	1	1	2	1	1	0	0^a^	0^a^	1	1	1	0^a^	0^a^	1	0	11	65
E	Benedetti et al. (35)	1	1	0	1	1	1	1	0^a^	0^a^	1	1	1	0	0^a^	0	0	9	53
F	Bolink et al. (36)	1	1	1	2	1	1	0	1	0^a^	1	1	0	0^a^	0^a^	1	0	11	65
G	Brand & Crowninshield (37)	1	1	0	1	0	0	0	0^a^	0^a^	1	0^a^	1	0^a^	0^a^	0	0	5	29
	[CON data: Crowninshield et al. (38)]																		
H	Constantinou et al. (39)	1	1	1	2	1	1	1	0^a^	1	1	1	1	1	1	0	1	15	88
	Diamond et al. (13)	1	1	1	2	1	1	0	0^a^	0^a^	1	1	1	0^a^	0^a^	0	0	10	59
I	Eitzen et al. (11)	1	1	1	2	1	1	1	1	1	1	1	1	0	0^a^	1	0	14	82
J	Foucher (40)	1	1	1	2	1	1	1	0^a^	0^a^	1	1	1	1	0^a^	1	0	13	76
K	Foucher et al. (41)	1	1	0	2	0	1	1	0^a^	0^a^	1	1	1	0^a^	0^a^	1	0	10	59
L	Foucher & Wimmer (42)	1	1	0	1	0	1	1	0^a^	0^a^	1	1	1	0^a^	0^a^	0	0	8	47
	Foucher et al. (43)	1	1	0	2	0	1	1	0^a^	0^a^	1	1	1	0^a^	0^a^	1	1	11	65
M	Hall et al. (44)	1	1	1	2	1	1	1	0^a^	0^a^	1	1	1	1	0^a^	0	0	12	71
N	Hara et al. (45) [CON data: Hara et al. (46)]	1	1	1	1	1	1	0	0^a^	0^a^	1	1	1	0^a^	0^a^	0	0	9	53
O	Hurwitz et al. (47)	1	0	0	2	1	1	1	0^a^	0^a^	0	1	1	0^a^	0^a^	1	0	9	53
P	Ismailidis et al. (48)	1	1	1	2	1	1	1	0^a^	0^a^	1	1	1	0^a^	0^a^	0	1	12	71
	Ismailidis et al. (49)	1	1	1	2	1	1	1	0^a^	0^a^	1	1	1	0	0^a^	0	1	12	71
	Nüesch et al. (50)	1	1	1	2	1	1	1	0^a^	0^a^	1	1	1	0^a^	0^a^	0	1	12	71
Q	Kataoka et al. (51)	1	1	1	2	1	0	1	0^a^	0^a^	1	1	1	0^a^	0^a^	1	0	11	65
R	Kubota et al. (52)	1	1	1	2	1	1	0	0^a^	0^a^	1	0	1	0^a^	0^a^	1	0	10	59
S	Kumar et al. (12)	1	1	1	2	1	1	1	0^a^	0^a^	1	1	1	1	0^a^	1	0	13	76
T	Leigh et al. (15)	1	1	1	2	1	1	1	0^a^	0^a^	1	1	1	0^a^	0^a^	1	1	13	76
U	Meyer et al. (53)	1	1	1	2	1	1	1	0^a^	0^a^	1	1	1	0	0^a^	1	0	12	71
	Wesseling et al. (54)	1	1	0	2	1	1	1	0^a^	0^a^	1	1	1	0^a^	0^a^	1	0	11	65
V	Ornetti et al. (55)	1	1	1	1	1	1	1	0^a^	0^a^	1	1	1	0^a^	0^a^	0	0	10	59
W	Popovic et al. (56)	1	1	1	2	1	1	1	0^a^	0	1	1	1	1	0^a^	1	0	13	76
X	Porta et al. (57)	1	1	1	2	1	1	1	0	0^a^	1	?	1	0	0^a^	1	0	12	71
Y	Reininga et al. (17)	1	1	1	2	1	1	1	0^a^	0^a^	1	1	1	0	0^a^	1	0	12	71
Z	Schmidt et al. (58)	1	1	1	2	1	1	1	0^a^	0^a^	1	1	1	0	0^a^	0	0	11	65
	Stief et al. (59)	1	1	1	2	1	1	1	0^a^	0^a^	1	1	1	0	0^a^	0	0	11	65
AA	Schmitt et al. (60)	1	1	1	2	1	1	1	0^a^	0^a^	1	1	1	0	0^a^	0	0	11	65
AB	Steingrebe et al. (61)	1	1	1	2	1	1	1	0	0^a^	1	1	1	0^a^	0^a^	1	0	12	71
AC	Tanaka (62)	0	1	0	1	0	0	0	0^a^	0^a^	0	0^a^	0	0^a^	0^a^	0	0	2	12
AD	Tateuchi et al. (63)	1	1	0	2	1	1	0	0^a^	0^a^	1	1	1	0^a^	0^a^	0	0	9	53
AE	Thurston (64)	1	1	0	1	1	1	0	0^a^	0^a^	1	0	1	0^a^	0^a^	0	0	7	41
Z & AF	van Drongelen et al. (65)	1	1	0	2	1	1	0	0^a^	0^a^	1	1	1	0	0^a^	0	0	9	53
	van Drongelen et al. (66)	1	1	0	2	1	1	1	0^a^	0^a^	1	1	1	0	0^a^	0	0	10	59
AF	van Drongelen et al. (67)	1	1	0	2	1	1	1	0^a^	0^a^	1	1	1	0^a^	0^a^	0	0	10	59
	van Drongelen et al. (68)	1	1	0	2	1	1	1	0^a^	0^a^	1	1	1	1	0^a^	0	0	11	65
AG	Watanabe et al. (69)	1	1	0	1	1	1	0	0^a^	0^a^	1	1	1	0^a^	0^a^	0	0	8	47
	Watanabe et al. (70)	1	1	0	1	1	1	0	0^a^	0^a^	1	1	1	0^a^	0^a^	0	0	8	47
AH	Watelain et al. (16)	1	1	1	2	1	1	0	0^a^	0^a^	1	1	1	0^a^	0^a^	0	0	10	59
AI	Zügner et al. (20)	1	1	1	2	1	1	1	0^a^	0^a^	1	1	1	0	0^a^	1	0	12	71

^a^ Unable to determine.

Most studies have high scores regarding reporting (Q1–3, 5–7, 10) and internal validity (bias, Q16, 18, 20). However, external validity (Q11, 12) is hardly ever to determine as detailed information about the recruitment process is missing. Likewise, questions regarding internal validity (confounding, Q21, 22) are often undetermined as information on CON subject recruitment and time period of subject recruitment is missing. Power analysis was only reported in 6 of the revised 47 reports (Q27).

### 3.2. Gait

The results from 33 studies analysing gait movement are reviewed below. The results are presented separated by joint, movement plane and laterality. Within each subsection, the results are presented in the following order: results of the meta-analysis, a qualitative summary of data and studies not included in the meta-analysis, results of continuous angle-time curve analyses and, if available, results of subgroup analyses.

#### 3.2.1. Hip joint

##### 3.2.1.1. Sagittal plane kinematics

###### Ipsilateral

**Figure 2:**
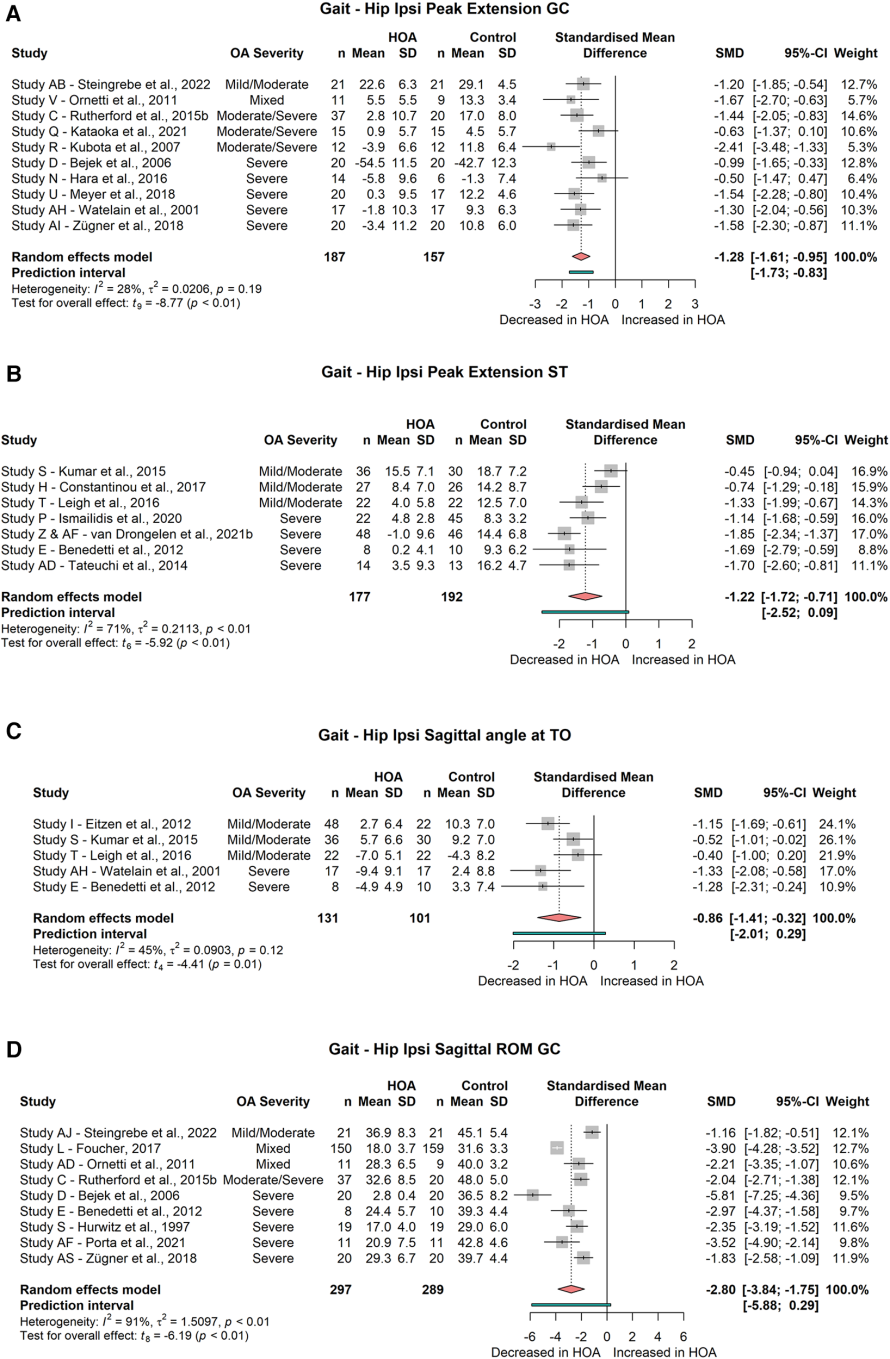
Forest plot of standardised and pooled effect sizes (random-effects model) with *I*^2^ heterogeneity statistics for: (**A**) ipsilateral peak hip extension during gait cycle (GC), (**B**) ipsilateral peak hip extension during stance phase (ST), (**C**) ipsilateral hip sagittal angle at toe-off (TO), (**D**) ipsilateral hip sagittal range of motion (ROM) across gait cycle; during gait.

Subjects with HOA have reduced peak extension of the affected hip joint during the gait cycle (GC) (10 studies, SMD = −1.28; 95% CI = −1.61, −0.95; *I*^2^ = 28; Figure 2A). Similar results were found for the peak hip extension during the stance phase (SP) (7 studies, SMD = −1.22; 95% CI = −1.72, −0.71; *I*^2^ = 71; Figure 2B). Hip sagittal angle at toe-off was significantly reduced (5 studies, SMD = −0.86; 95% CI = −1.41, −0.32; *I*^2^ = 45; Figure 2C). Peak flexion was not different between HOA and CON subjects (GC: 8 studies, SMD = −0.53; 95% CI = −1.25, 0.19; *I*^2^ = 80; SP: 5 studies, SMD = −0.36; 95% CI = −1.34, 0.62; *I*^2^ = 86; swing phase: 2 studies, SMD = −1.26; 95% CI = −6.81, 4.28; *I*^2^ = 54).

Hip flexion at initial contact did not differ between subject groups (3 studies, SMD = −0.67; 95% CI = −2.09, 0.76; *I*^2^ = 76).

There was a significant reduction of the sagittal hip range of motion (ROM) across the gait cycle (9 studies, SMD = −2.80; 95% CI = −3.84, −1.75; *I*^2^ = 91, Figure 2D), but not across the stance phase (5 studies, SMD = −1.42; 95% CI = −3.15, 0.31; *I*^2^ = 95).

Studies not included in the meta-analysis also showed reduced peak hip extension (60, 62). Results on peak hip flexion varied, with Schmitt et al. (60) reporting increased peak hip flexion in unilateral subjects and Tanaka (62) in bilateral subjects. In contrast, Tanaka (62) showed reduced peak hip flexion in unilateral HOA subjects. Additionally, a reduction of hip flexion at initial contact was found (60). Reduced sagittal hip ROM was found in 3 out of 4 studies [significant reduction in ROM during SP (41, 42) and swing phase (49); no significant difference in ROM during GC (37)].

All 6 of the studies [(31), study *P* (48, 50), study *U* (53, 54) , 57, study Z & AF (65, 66)] analysing continuous sagittal hip angle-time curves show differences between HOA and CON subjects.

Subgroup analysis did not find significant differences in peak hip flexion during the stance phase or gait cycle for subjects with mild/moderate HOA (ST: 2 studies, SMD = 0.31; 95% CI = −4.33, 4.95; *I*^2^ = 86; GC: 2 studies, SMD = −0.36; 95% CI = −1.53, 0.82 *I*^2^ = 0) or subjects with severe HOA (ST: 3 studies, SMD = −0.80; 95% CI = −2.28, 0.67; *I*^2^ = 80; GC: 4 studies, SMD = −0.61; 95% CI = −2.61, 1.39; *I*^2^ = 85).

**Figure 3:**
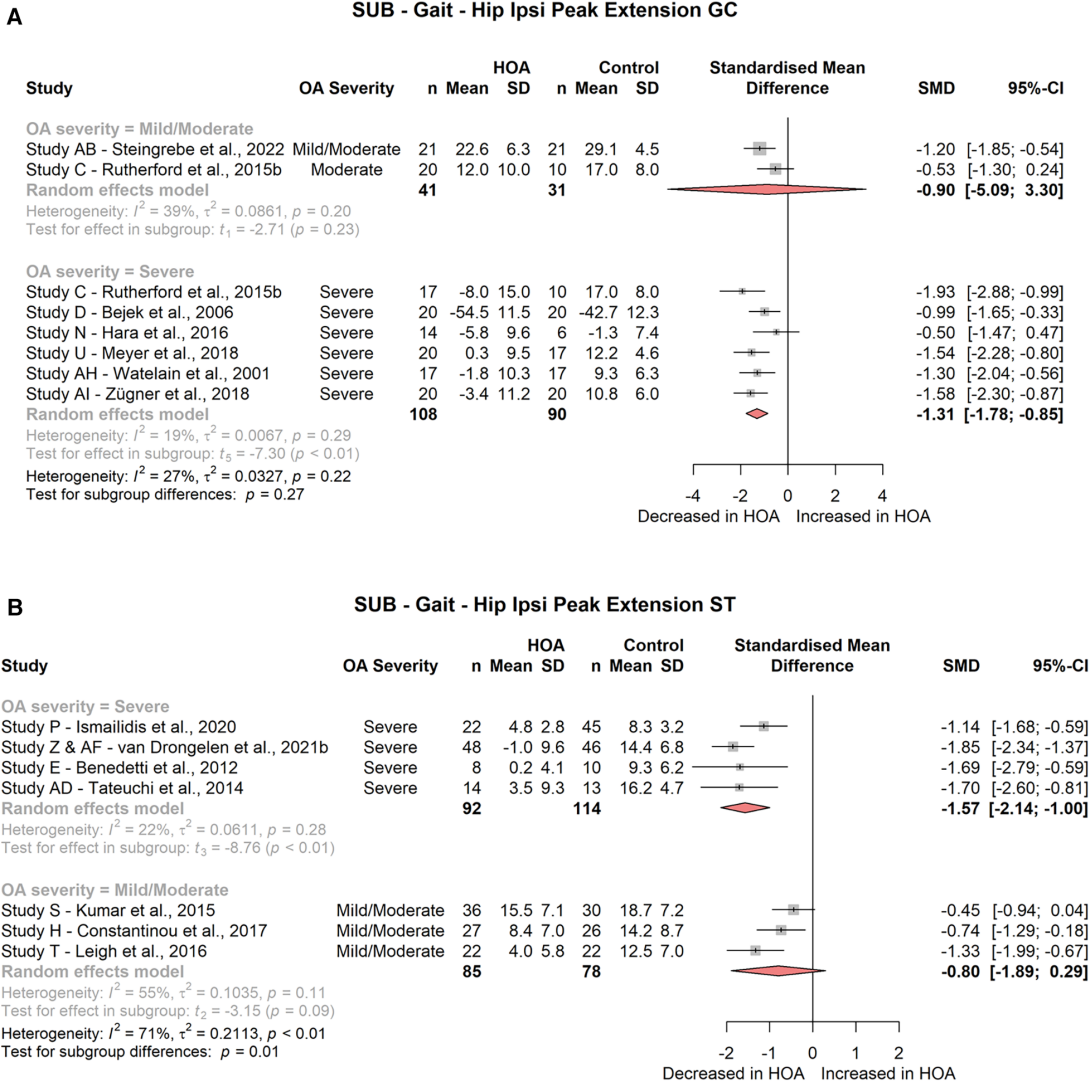
Forest plot of standardised and pooled effect sizes (random-effects model) with *I*^2^ heterogeneity statistics for subgroup analyses on: (**A**) ipsilateral peak hip extension during gait cycle (GC), (**B**) ipsilateral peak hip extension during stance phase (ST); during gait.

Hip peak extension during the stance phase or gait cycle was significantly different for subjects with severe HOA (ST: 4 studies, SMD = −1.57; 95% CI = −2.14, −1.00; *I*^2^ = 22; GC: 6 studies, SMD = −1.31; 95% CI = −1.78, −0.85; *I*^2^ = 19, Figure 3) but not for subjects with mild/moderate HOA (ST: 3 studies, SMD = −0.80; 95% CI = −1.89, 0.29; *I*^2^ = 55; GC: 2 studies, SMD = −0.90; 95% CI = −5.09, 3.30; *I*^2^ = 39).

**Figure 4:**
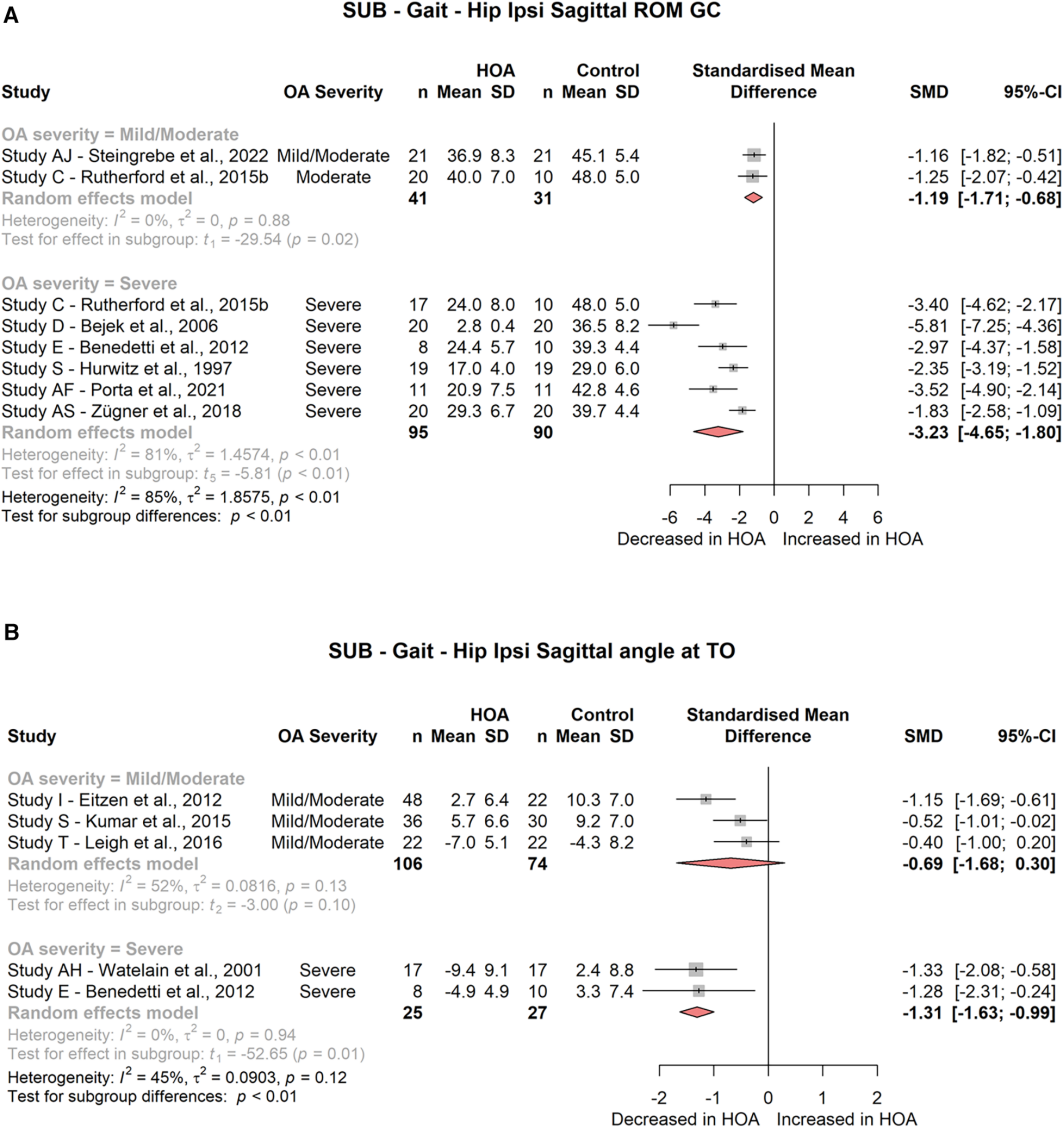
Forest plot of standardised and pooled effect sizes (random-effects model) with *I*^2^ heterogeneity statistics for subgroup analyses on: (**A**) ipsilateral hip sagittal range of motion (ROM) across gait cycle (GC), (**B**) ipsilateral hip sagittal angle at toe-off (TO); during gait.

Hip sagittal angle at toe-off was significantly decreased in severe HOA subjects (2 studies, SMD = −1.31; 95% CI = −1.63, −0.99; *I*^2^ = 0, Figure 4B), but not in mild/moderate HOA subjects (3 studies, SMD = −0.69; 95% CI = −1.68, 0.30; *I*^2^ = 52).

Hip sagittal ROM across the gait cycle was significantly decreased for mild/moderate (2 studies, SMD = −1.19; 95% CI = −1.71, −0.68; *I*^2^ = 0) and severe HOA (6 studies, SMD = −3.23; 95% CI = −4.65, −1.80; *I*^2^ = 81) subjects (Figure 4A). Hip sagittal ROM across the stance phase was not different for any of the HOA severity subgroups (mild/moderate: 3 studies, SMD = −0.70; 95% CI = −2.85, 1.45; *I*^2^ = 91; severe: 2 studies, SMD = −2.51; 95% CI = −16.52, 11.51; *I*^2^ = 96).

###### Contralateral

**Figure 5:**
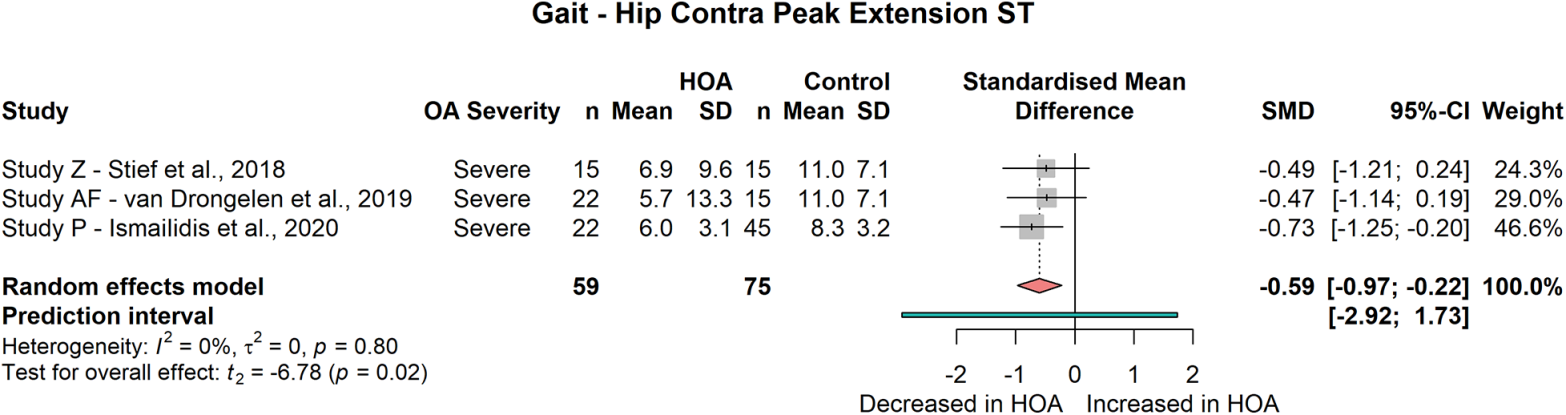
Forest plot of standardised and pooled effect sizes (random-effects model) with *I*² heterogeneity statistics for contralateral peak hip extension during stance phase (ST); during gait.

Subjects with unilateral HOA also displayed a reduced peak extension of the contralateral hip joint during the stance phase of gait (3 studies, SMD = −0.59; 95% CI = −0.97, −0.22; *I*^2^ = 0, Figure 5) but not during the gait cycle (2 studies, SMD = −0.15; 95% CI = −7.76, 7.45; *I*^2^ = 78). No differences between HOA and CON subjects were found for contralateral peak hip flexion during the stance phase (3 studies, SMD = 0.23; 95% CI = −0.41, 0.87; *I*^2^ = 0) or gait cycle (2 studies, SMD = −0.7; 95% CI = −2.38, 0.98; *I*^2^ = 0), nor for contralateral sagittal hip ROM across the stance phase (3 studies, SMD = −0.3; 95% CI = −0.74, 0.15; *I*^2^ = 0) or gait cycle (4 studies, SMD = −0.79; 95% CI = −2.65, 1.06; *I*^2^ = 88) during the meta-analysis.

A study not included in the meta-analysis also reported reduced contralateral peak hip extension and increased peak hip flexion angles (62).

Of the 4 studies (50, 54, 57, 65) analysing contralateral sagittal hip angle-time curves, 3 found differences in contralateral hip sagittal angles.

##### 3.2.1.2. Frontal plane kinematics

###### Ipsilateral

**Figure 6:**
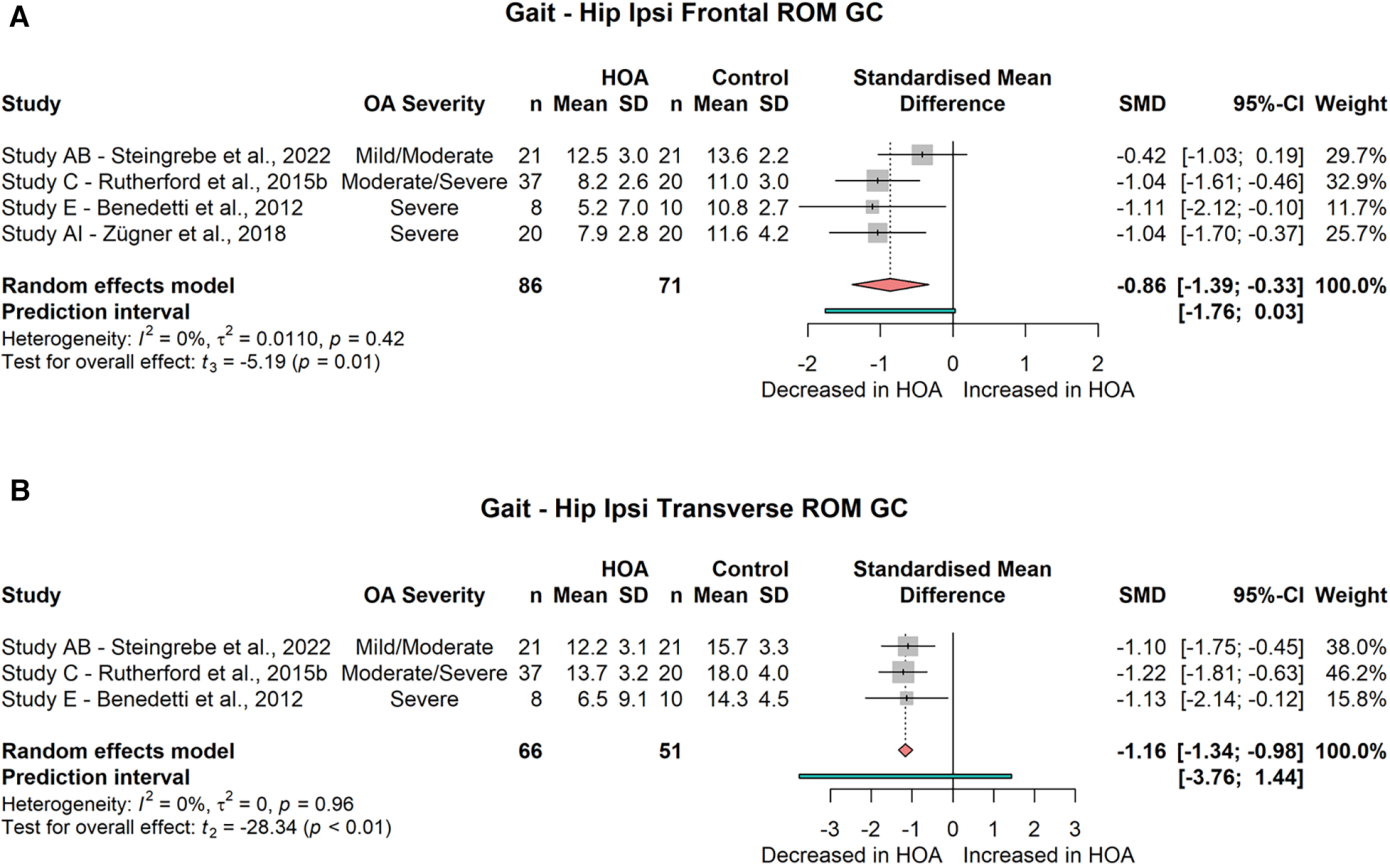
Forest plot of standardised and pooled effect sizes (random-effects model) with *I*^2^ heterogeneity statistics for (**A**) ipsilateral hip frontal range of motion (ROM) across gait cycle (GC), (**B**) ipsilateral hip transverse ROM across GC; during gait.

The meta-analysis showed no significant differences for peak hip abduction during the gait cycle (4 studies, SMD = −0.7; 95% CI = −1.83, 0.44; *I*^2^ = 75) or at toe-off (2 studies, SMD = −0.16; 95% CI = −5.24, 4.92; *I*^2^ = 66). Peak hip adduction did not differ significantly between groups either during the gait cycle or during stance (GC: 5 studies, SMD = −0.35; 95% CI = −0.87, 0.18; *I*^2^ = 42; ST: 4 studies, SMD = −0.43; 95% CI = −1.24, 0.38; *I*^2^ = 46), but a significant reduction of the frontal plane hip ROM across the gait cycle (4 studies, SMD = −0.86; 95% CI = −1.93, −0.33; *I*^2^ = 0, Figure 6A) was found. Frontal plane hip ROM across the stance phase was not different between groups (2 studies, SMD = −1.38; 95% CI = −11.65, 8.88; *I*^2^ = 88).

Studies not included in the meta-analysis showed an increased hip abduction at midstance (15), as well as decreased peak hip adduction during early and late stance (13). Peak abduction during stance (63) and swing (35), and hip frontal angle at peak hip extension (15), did not differ between subject groups.

Both studies using SPM analysis found differences in hip frontal plane angle [study *U* (53, 54), 65]. Van Drongelen et al. (65) found differences for subjects with unilateral HOA but not for those with bilateral HOA.

Results from the subgroup analysis did not find group differences in peak hip abduction or adduction during the gait cycle for mild/moderate HOA subjects (abduction: 2 studies, SMD = −0.01; 95% CI = −2.05, 2.03; *I*^2^ = 0; adduction: 2 studies, SMD = −0.44; 95% CI = −2.80, 1.91; *I*^2^ = 0) or for severe HOA subjects (abduction: 2 studies, SMD = −0.94; 95% CI = −7.33, 5.46; *I*^2^ = 70; adduction: 2 studies, SMD = −0.02; 95% CI = −7.02, 6.98; *I*^2^ = 78).

Hip frontal ROM across the gait cycle was not different in mild/moderate (2 studies, SMD = −0.39; 95% CI = −0.93, 0.16; *I*^2^ = 0) or in severe HOA subjects (2 studies, SMD = −1.35; 95% CI = −2.74, 0.04; *I*^2^ = 37).

###### Contralateral

Van Drongelen et al. (67, 68) did not find a significant difference between CON and HOA subjects for the peak adduction angle during stance.

Results from two SPM analyses (54, 65) both show differences in contralateral hip frontal angle-time curves.

##### 3.2.1.3. Transverse plane kinematics

###### Ipsilateral

The meta-analysis yielded no difference for the peak external rotation angle (2 studies, SMD = 0.17; 95% CI = −0.59, 0.94; *I*^2^ = 0) or the peak internal rotation angle during the gait cycle (3 studies, SMD = −0.5; 95% CI = −1.75, 0.76; *I*^2^ = 59). Also, the transverse angle at toe-off was not different (2 studies, SMD = −0.11; 95% CI = −14.61, 14.39; *I*^2^ = 95). However, a significantly decreased transverse ROM across the gait cycle (3 studies, SMD = −1.16; 95% CI = −1.34, −0.98; *I*^2^ = 0, Figure 6B) was found.

Data not included in the meta-analysis showed a significant reduction of the peak internal rotation angle during the stance phase and a significant increase in peak external rotation during the swing phase (35). Similarly, Leigh and colleagues (15) found the hip joint of HOA subjects was significantly more externally rotated at terminal hip extension but not at midstance.

One study (54) found differences in the hip transverse angle-time curve in an SPM analysis.

The subgroup analysis did not show significant differences in hip transverse ROM across the gait cycle for mild/moderate (2 studies, SMD = −0.84; 95% CI = −4.60, 2.93; *I*^2^ = 26) or severe HOA subjects (2 studies, SMD = −1.47; 95% CI = −5.39, 2.45; *I*^2^ = 0).

###### Contralateral

Only 1 study (54) reported data on transverse plane contralateral hip angles and did not find any significant differences during an SPM analysis.

#### 3.2.2. Knee joint

##### 3.2.2.1. Sagittal plane kinematics

###### Ipsilateral

**Figure 7:**
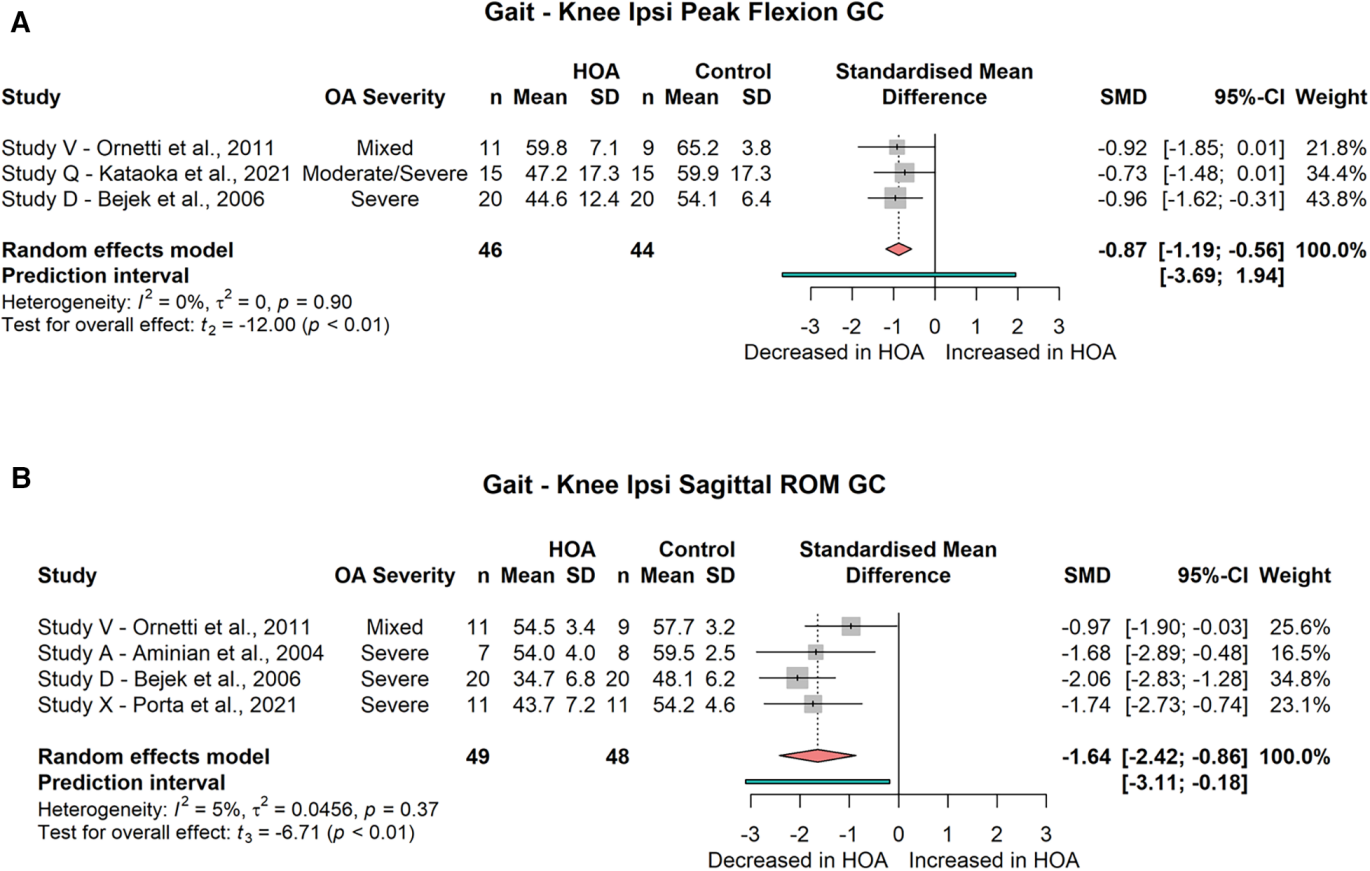
Forest plot of standardised and pooled effect sizes (random-effects model) with *I*^2^ heterogeneity statistics for: (**A**) ipsilateral knee peak flexion during gait cycle (GC), (**B**) ipsilateral knee sagittal range of motion (ROM) across GC; during gait.

The meta-analysis did not show a significant difference for peak knee extension during the gait cycle (3 studies, SMD = 0.72; 95% CI = −2.21, 3.65; *I*^2^ = 87) or stance phase (2 studies, SMD = 0.81; 95% CI = −13.61, 15.23; *I*^2^ = 96). Peak flexion was significantly reduced during the gait cycle (3 studies, SMD = −0.87; 95% CI = −1.19, −0.56; *I*^2^ = 0, Figure 7A), but not during stance (3 studies, SMD = −0.68; 95% CI = −1.55, 0.2; *I*^2^ = 44). Sagittal knee angle at initial contact (2 studies, SMD = −0.09; 95% CI = −0.54, 0.37; *I*^2^ = 0), midstance (2 studies, SMD = 0.22; 95% CI = −4.26, 4.69; *I*^2^ = 68), toe-off (2 studies, SMD = 0.22; 95% CI = −1.60, 2.03; *I*^2^ = 0) and peak hip extension (2 studies, SMD = 1.30; 95% CI = −0.52, 3.11; *I*^2^ = 0) was not different between groups. Sagittal knee ROM was significantly reduced across the gait cycle (4 studies, SMD = −1.64; 95% CI = −2.24, −0.86; *I*^2^ = 5, Figure 7B) but not across the stance phase (3 studies, SMD = −1.06; 95% CI = −5.25, 3.13; *I*^2^ = 97).

A study not included in the meta-analysis by Ismailidis et al. (49), described a significantly decreased knee ROM across the swing phase, while peak flexion during swing did not differ between groups.

Analyses of knee sagittal angle-time curves showed differences in all 5 studies [31, study *P* (48, 50) 54, 57, study Z & AF (65, 66)].

###### Contralateral

**Figure 8:**
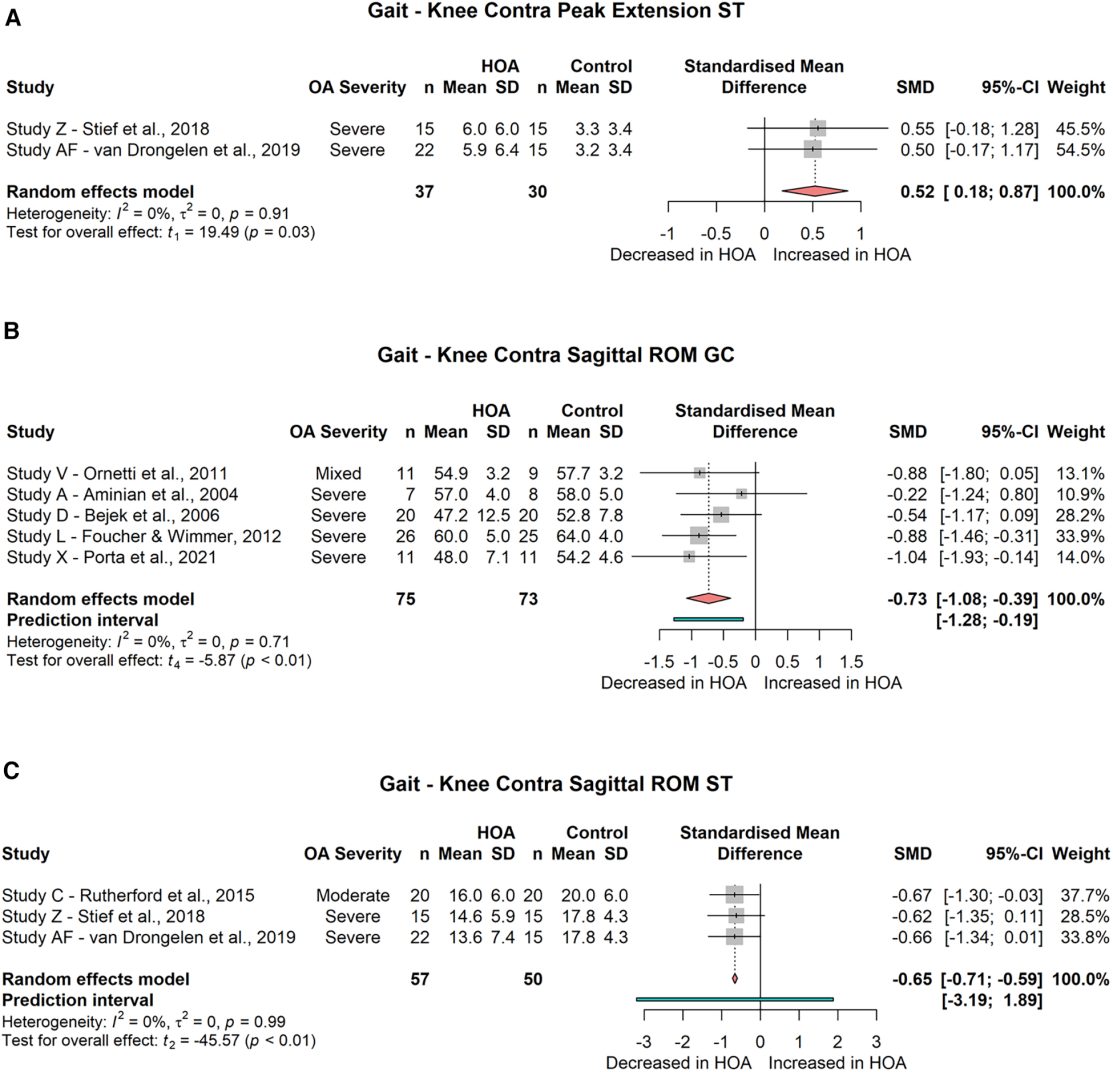
Forest plot of standardised and pooled effect sizes (random-effects model) with *I*^2^ heterogeneity statistics for: (**A**) contralateral knee peak extension during stance phase (ST), (**B**) contralateral knee sagittal range of motion (ROM) across gait cycle (GC), (**C**) contralateral knee sagittal ROM across ST; during gait.

For the contralateral knee joint, the meta-analysis did not show a significant difference for peak knee extension during the gait cycle (2 studies, SMD = −0.03; 95% CI = −1.77, 1.71; *I*^2^ = 0) but a significant difference during the stance phase (2 studies, SMD = 0.52; 95% CI = 0.18, 0.87; *I*^2^ = 0, Figure 8A). Peak flexion was not significantly reduced during the gait cycle (2 studies, SMD = −0.87; 95% CI = −3.21, 1.47; *I*^2^ = 0) or stance phase (3 studies, SMD = −0.05; 95% CI = −0.66, 0.55; *I*^2^ = 0). Sagittal knee ROM was significantly reduced across both the gait cycle (5 studies, SMD = −0.73; 95% CI = −1.08, −0.39; *I*^2^ = 0, Figure 8B) and the stance phase (3 studies, SMD = −0.65; 95% CI = −0.71, −0.59; *I*^2^ = 0, Figure 8C).

Out of 4 studies analysing sagittal contralateral knee angles time-curves, 2 found differences between subject groups (54, 57) and 2 did not (50, 65).

##### 3.2.2.2. Frontal plane kinematics

###### Ipsilateral

The meta-analysis did not show a difference between groups for the ROM across the stance phase (2 studies, SMD = −0.09; 95% CI = −5.55, 5.37; *I*^2^ = 68).

One study not included in the meta-analysis reported no significant group differences for the frontal knee angle at midstance, toe-off or peak hip extension (15).

###### Contralateral

The meta-analysis did not show a difference between groups for the ROM across the stance phase (2 studies, SMD = −0.08; 95% CI = −5.67, 5.51; *I*^2^ = 69).

##### 3.2.2.3. Transverse plane kinematics

###### Ipsilateral

Rutherford et al. (14) did not find a significant difference in transverse knee ROM across the stance phase. However, Leigh et al. (15) found significantly increased external knee rotation angles at midstance and at peak hip extension, but not at toe-off.

###### Contralateral

No studies were retrieved that presented data on contralateral transverse plane knee kinematics.

#### 3.2.3. Ankle joint

##### 3.2.3.1. Sagittal plane kinematics

###### Ipsilateral

The meta-analysis did not show differences for the peak dorsiflexion angle during the gait cycle (2 studies, SMD = −0.01; 95% CI = −9.38, 9.37; *I*^2^ = 82) or stance phase (2 studies, SMD = −0.62; 95% CI = −3.77, 5.00; *I*^2^ = 54). Peak plantar flexion during the gait cycle (3 studies, SMD = 0.46; 95% CI = −0.98, 1.90; *I*^2^ = 51) and during the stance phase (2 studies, SMD = −0.09; 95% CI = −3.67, 3.49; *I*^2^ = 35) was not different between groups. Ankle sagittal ROM across the gait cycle (2 studies, SMD = −0.53; 95% CI = −6.47, 5.41; *I*^2^ = 53) and across the stance phase (2 studies, SMD = 0.34; 95% CI = −1.61, 2.29; *I*^2^ = 0) did not differ between groups. The ankle angles at initial contact (2 studies, SMD = 0.07; 95% CI = −2.45, 2.58; *I*^2^ = 25), midstance (2 studies, SMD = 0.18; 95% CI = −1.24, 1.60; *I*^2^ = 0), toe-off (2 studies, SMD = 0.02; 95% CI = −1.42, 1.47; *I*^2^ = 0) and peak hip extension (2 studies, SMD = 0.25; 95% CI = −1.72, 2.21; *I*^2^ = 0) were not different between groups.

In 5 studies ankle sagittal angle-time curves were analysed. Although 2 studies found differences between groups (54, 57), 2 did not (31, 65) and 1 study yielded contradicting results in 2 reports [study *P* (48, 50)].

###### Contralateral

Ankle angle ROM across the gait cycle was not different between groups (2 studies, SMD = −0.47; 95% CI = −3.88, 2.94; *I*^2^ = 0).

Data not included in the meta-analysis showed differences in peak dorsiflexion angle during the gait cycle (*p* = 0.05) but not in peak plantar flexion angle (*p* = 0.087) (55).

In 4 studies contralateral sagittal ankle angle-time curves were analysed. Three of these studies found differences between groups (50, 54, 57) but 1 study did not (65).

##### 3.2.3.2. Frontal plane kinematics

###### Ipsilateral

Only 1 study analysed frontal plane ankle angles, and found a reduced ankle inversion at toe-off. Frontal ankle angle at midstance and at peak hip extension did not differ between groups (15).

###### Contralateral

No studies were retrieved that presented data on contralateral frontal plane ankle kinematics.

##### 3.2.3.3. Transverse plane kinematics

###### Ipsilateral

Only 1 study analysed transverse plane ankle kinematics and did not find differences in ankle angles at midstance, toe-off or peak hip extension (15).

###### Contralateral

No studies were retrieved that presented data on contralateral transverse plane ankle kinematics.

#### 3.2.4. Pelvis

##### 3.2.4.1. Sagittal plane kinematics

The meta-analysis found no differences in peak anterior tilt during either the gait cycle (4 studies, SMD = 0.70; 95% CI = −0.11, 1.51; *I*^2^ = 27) or the stance phase (2 studies, SMD = 0.68; 95% CI = −5.07, 6.43; *I*^2^ = 85). Likewise, pelvis peak posterior tilt did not differ between groups during the gait cycle (3 studies, SMD = 0.72; 95% CI = −1.78, 3.23; *I*^2^ = 79). Pelvis angle at toe-off also did not differ between CON and HOA subjects (2 studies, SMD = −0.38; 95% CI = −12.24, 11.49; *I*^2^ = 93).

Pelvis ROM across the gait cycle was not different between groups (3 studies, SMD = 1.85; 95% CI = −1.99, 5.69; *I*^2^ = 91).

Studies not included in the meta-analysis found a significantly increased anterior pelvis tilt at peak hip extension (15), but not at midstance (15, 69) initial contact or toe-off (69) in HOA subjects.

One study analysed pelvis sagittal angle using SPM analysis and found differences between bilateral HOA and CON subjects but not between unilateral HOA and CON subjects (65).

##### 3.2.4.2. Frontal plane kinematics

The meta-analysis found no differences in peak inferior obliquity during the gait cycle (2 studies, SMD = 2.25; 95% CI = −12.44, 16.93; *I*^2^ = 92). Peak superior obliquity was not different in the gait cycle (2 studies, SMD = −0.28; 95% CI = −7.83, 7.27; *I*^2^ = 80) or in the stance phase (3 studies, SMD = −0.43; 95% CI = −2.53, 1.61; *I*^2^ = 78). Likewise, pelvic frontal angle at toe-off did not differ between groups (2 studies, SMD = −0.53; 95% CI = −8.60, 7.54; *I*^2^ = 86).

Pelvis frontal plane ROM was not different between groups either across the gait cycle (5 studies, SMD = −0.19; 95% CI = −3.33, 2.96; *I*^2^ = 96) or in the stance phase (2 studies, SMD = −0.31; 95% CI = −6.80, 6.18; *I*^2^ = 81).

Studies not included in the meta-analysis found no differences in peak inferior obliquity during the swing phase (35); or for the pelvis frontal angle at peak hip extension (15), initial contact, midstance or toe-off [study AG (69, 70)].

Differences between groups were found for pelvis frontal angle at midstance (15) and peak inferior obliquity during single-limb stance (67).

One study analysed pelvis frontal angle using SPM analysis and found differences between bilateral HOA and CON subjects but not between unilateral HOA and CON subjects (65).

##### 3.2.4.3. Transverse plane kinematics

The meta-analysis did not show differences in the pelvis transverse ROM across the gait cycle (3 studies, SMD = −0.06; 95% CI = −0.44, 0.32; *I*^2^ = 0) or the pelvis transverse angle at toe-off (2 studies, SMD = 0.35; 95% CI = −3.13, 3.82; *I*^2^ = 29).

Studies not included in the meta-analysis found no differences between groups for the transverse pelvic ROM across the stance phase (16), pelvis angle at peak hip extension (15) or peak posterior rotation (35). However, one study reported significant group differences for the pelvis transverse angle at midstance (15).

### 3.3. Stair walking

Two studies analysing stair walking are reviewed below, separated into stair ascent and stair descent.

#### 3.3.1. Stair ascent

Peak ipsilateral hip flexion, adduction and internal rotation during stance phase did not differ between groups (flexion: 2 studies, SMD = −0.49; 95% CI = −9.71, 8.73; *I*^2^ = 90; adduction: 2 studies, SMD = −0.17; 95% CI = −8.42, 8.08; *I*^2^ = 88; internal rotation: 2 studies, SMD = 0.41; 95% CI = −3.03, 3.85; *I*^2^ = 38).

Results not included in the meta-analysis showed no differences in peak hip extension during the gait cycle (44) or during the stance phase (56). Peak flexion was significantly reduced in the swing phase (44). Hip sagittal ROM across the gait cycle was significantly reduced (44).

Hip peak abduction was significantly reduced during swing (44) and stance phase (56). Hip frontal ROM was significantly reduced (44).

Peak external rotation was significantly reduced during stance phase (56) but not during swing phase (44). Transverse hip ROM was significantly reduced (44).

Peak ipsilateral knee angles in the sagittal and frontal planes during the stance phase did not differ between groups (56). Likewise, peak knee internal rotation was not different. However, peak knee external rotation was significantly increased in HOA subjects.

Peak ipsilateral sagittal and frontal ankle angles did not differ between groups (56). However, peak ankle internal rotation was significantly reduced while peak external rotation was significantly increased in HOA subjects (56).

Peak contralateral pelvis inferior and superior obliquity did not differ between groups (44).

#### 3.3.2. Stair descent

Peak ipsilateral hip flexion during stance did not differ between groups (2 studies, SMD = −0.06; 95% CI = −1.36, 1.25; *I*^2^ = 0). Likewise, peak hip adduction and abduction did not differ between CON and HOA subjects (adduction: 2 studies, SMD = 0.05; 95% CI = −5.10, 5.21; *I*^2^ = 71; abduction: 2 studies, SMD = −0.03; 95% CI = −5.47, 5.42; *I*² = 74). Peak internal and external hip rotation were not different between groups (internal rotation: 2 studies, SMD = 0.15; 95% CI = −4.21, 4.52; *I*² = 60; external rotation: 2 studies, SMD = −0.37; 95% CI = −1.42, 0.69; *I*² = 0).

Results not included in the meta-analysis showed no differences in peak hip extension during either the stance phase (56) or gait cycle (44). Likewise, peak flexion during swing was not different between groups (44). However, sagittal hip ROM was significantly reduced in HOA subjects (44).

There was no difference between groups in peak hip abduction during swing, hip frontal plane ROM across the gait cycle (44) or transverse plane hip ROM (44).

While peak knee flexion did not differ between groups, peak knee extension was significantly increased in HOA subjects (56).

Frontal plane peak knee angles did not differ between groups (56).

Transverse plane knee angles showed significantly reduced peak internal and significantly increased peak external rotation (56).

At the ankle joint, HOA subjects showed significantly increased peak plantar flexion. Peak dorsi flexion did not differ between groups (56), and neither did peak ankle eversion and inversion (56).

Ankle peak internal rotation was significantly reduced while peak external rotation was significantly increased in HOA subjects (56).

Peak contralateral pelvis inferior and superior obliquity did not differ between groups (44).

### 3.4. Turning while walking

In a study by Tateuchi and colleagues (63), 45° turns conducted either in a step turn or in a spin turn manner were analysed in subjects with severe HOA. Peak angles during the stance phase were described.

During the step turn, significantly decreased peak hip flexion and extension were found. Peak hip abduction was also significantly reduced. No differences were found for peak hip adduction.

Sagittal plane peak angles of the knee and ankle joint did not differ between groups.

During the spin turn, significantly decreased peak hip flexion and extension were found. Peak hip abduction did not differ between groups. However, significantly reduced peak hip adduction was found in HOA subjects.

Peak knee extension did not differ between groups but peak knee flexion was significantly reduced in HOA subjects.

Ankle sagittal peak angles did not differ between groups.

## 4. Discussion

The aim of this review and meta-analysis was to summarise existing literature on lower-limb joint kinematics during locomotion in subjects with HOA compared to healthy controls. Where possible, a meta-analysis was performed with the focus on HOA severity and uni- or bilateral involvement.

Overall, 47 reports from 35 individual studies were reviewed. The total score regarding risk of bias and quality of reporting of the included studies varied strongly, with older reports tending to show lower scores.

The first outcome of this systematic literature review is that studies on locomotion tasks other than gait are rare, with only 2 studies on stair walking (44, 56) and 1 study on curve walking (63).

Secondly, a large portion of the analysed subjects are classified as having severe HOA as well as unilateral HOA. This observation might originate in the fact that HOA subjects are often recruited prior to total hip arthroplasty and studies aim at evaluating rehabilitation after surgery. Due to the small number of studies with subjects with mild or moderate HOA, subgroup analyses for HOA severity were only possible for some parameters of ipsilateral hip kinematics. Likewise, it was not possible to perform subgroup analysis regarding HOA laterality, as for none of the revised parameters, data of at least 2 studies for each subgroup, namely unilateral and bilateral HOA subjects, were available. This was mainly caused by a lack of bilateral HOA subject groups or the unavailability of laterality information. As no conjoint analysis was possible, insights on the impact of laterality still have to be based on individual study results, such as those of Tanaka (62) or van Drongelen et al. (65).

Generally, it has to be noted that although 33 individual studies were included on gait, a conjoint analysis is hindered by the multitude of calculated parameters. For example, calculating peak angles or ROM across the stance phase is not comparable to the same parameters calculated across the entire gait cycle. Therefore, of the 68 combined analyses calculated for parameters on gait kinematics, only 10 include 5 or more individual studies. Thereof, 7 refer to ipsilateral hip sagittal angles, 1 refers to ipsilateral hip frontal angle, contralateral knee sagittal angle and pelvis frontal angle, respectively.

In 5 studies continuous angle-time curves were analysed by means of an SPM analysis [31, study *P* (48, 50), study *U* (53, 54), 65] or by point-by-point analysis (57). While these approaches can be advantageous, especially during explorative data analysis, the aggregation of results across multiple studies is difficult.

Five studies assessed kinematics using IMU sensors [17, Study *P* (48–50), 30, 36, 51]. Most of these studies focused on sagittal hip, knee and ankle angles or frontal pelvis angles and demonstrated significant group differences. Only 1 study used IMUs on frontal and transverse hip angles (51), but did not find significant differences.

### 4.1. Effects of hip osteoarthritis on gait kinematics

For the gait movement, the main results for the hip joint were an overall reduced peak extension in HOA subjects. However, during the subgroup analysis, only subjects with severe HOA demonstrated significant group differences. Similar results were found for the hip sagittal angle at toe-off, which makes sense as peak hip extension and toe-off occur very close to each other.

Reduction of the sagittal hip ROM across the gait cycle occurred with a very large effect size, and was present in subjects with both mild/moderate as well as severe HOA. In contrast, sagittal hip ROM across the stance phase was not different between groups. Thus, it might be crucial to capture stance and swing phase kinematics to discover deviations in gait caused by HOA.

Interestingly, peak hip extension was also reduced in the contralateral limb in subjects with severe unilateral HOA. As hip extension is closely connected to step length (71), this contradicts the consideration of increased step length in the contralateral limb to compensate for decreased ipsilateral step length (18). However, this may be because in most studies healthy CON subjects are not evaluated radiographically so structural changes in the contralateral limb cannot be excluded.

Frontal as well as transverse plane hip ROMs were reduced across the gait cycle. As neither of the peak angles demonstrated significant group differences, the ROM might be more sensitive to group differences as it captures changes occurring at both extrema of the dynamic movement. Little is known about frontal and transverse hip kinematics of the contralateral limb; however, in two studies analyses of contralateral frontal hip angle-time curves found differences between HOA and CON subjects, which requires further investigation.

Ipsilateral as well as contralateral sagittal plane knee kinematics are influenced by HOA. The ipsilateral limb shows decreased peak flexion and a reduced sagittal ROM across the gait cycle. The contralateral limb demonstrates increased peak extension, but with negligible effect size. However, sagittal knee ROM across the stance phase and gait cycle was significantly decreased with small to moderate effect size.

Very few studies analysed the effect of HOA on knee frontal and transverse kinematics. Although no differences were found for frontal plane knee angles, individual study results on knee transverse kinematics varied. Some studies found increased foot-progression angles during walking for subjects with HOA (58, 63), which might be linked to changes in knee rotation.

The meta-analysis did not yield any significant differences in ipsi- or contralateral sagittal ankle kinematics. However, individual study results as well as analysis of angle-time curves partly yielded significant group differences. Thus, ankle kinematics might only differ in specific settings or groups: this should be considered in future studies.

For the frontal and transverse planes, no data exist regarding contralateral ankle kinematics. Ipsilateral ankle kinematics in those planes were only analysed in 1 study which only found a reduced ankle inversion at toe-off.

The meta-analysis did not yield any significant differences in pelvis sagittal, frontal or transverse movement. However, it has to be noted that large study heterogeneity was observed for all analysed parameters, especially for the sagittal and frontal plane. For example, in the analysis of the frontal pelvis ROM across the gait cycle, the results from Bejek et al. (34) differed dramatically from those of the other studies and, if excluded, the random-effects model approach statistical significance (*p* = 0.06). One possible explanation for this deviation might come from different measurement techniques, as Bejek and colleagues were the only ones to use an ultrasound-based motion capture system. Peak anterior pelvis tilt approached significance (*p* = 0.07) in the meta-analysis with a moderate effect size. Anterior tilting of the pelvis might allow the patients to increase step length despite limitations in hip extension (15, 64). Our subgroup analysis did not show a significant difference in peak hip extension angle for subjects with mild or moderate HOA. Thus, compensatory pelvic motion might not or only to a limited extent be necessary in this subject group. Yet, this has to be evaluated in future studies.

The results from our analyses show that modifications of kinematic patterns are not limited to the ipsilateral side nor the affected joint but rather are a complex interplay of changes occurring at the pelvis and both lower limbs. These results are in line with those from whole-body analyses that show the highest discriminatory capacity in hip, knee and ankle sagittal angles and partly frontal plane ankle angles (31, 72, 73).

### 4.2. Effects of hip osteoarthritis on stair walking kinematics

For stair ascent, no significant differences between HOA and CON subjects were found during the meta-analysis. However, individual study results found decreased hip peak flexion during swing, causing a decrease in sagittal hip ROM. Stair ascent requires a greater sagittal hip ROM than level walking, and a high peak flexion is crucial for ensuring step clearance and avoidance of tripping (74). Thus, decreased peak flexion and sagittal ROM might make HOA subjects prone to falling during stair ascent. Likewise, peak abduction and external rotation of the hip were reduced, causing reduced hip ROM in the frontal and transverse planes. In contrast, the knee and ankle joints seem to be more externally rotated. Meyer et al. (75) reported reduced peak adduction during stair ascent as a strategy for a wider base of support, which was not present in our meta-analysis. Thus, adopting a toe-out gait by externally rotating the foot and tibia might be another strategy to broaden the area of support for the stance limb (76).

For stair descent, the meta-analysis did not yield any significant differences in hip peak angles. However, an individual study still reported reduced sagittal hip ROM (44).

Additionally, an increase in peak knee extension as well as peak plantar flexion was observed in 1 study (56). This might stem from an attempt to prolong contact with the stair at toe-off or to contact the stair sooner to reduce single-support time. Yet, this is speculative and warrants further investigation.

As in stair ascent, a more pronounced external rotation of the knee and ankle joints was observed.

Overall, it has to be considered that only two studies analysed stair walking in HOA subjects, thus insights are still very limited.

Additionally, the staircases used during the studies contained two or four steps, with the force plate included in the first step. Hence, the analysed step always contained movement initiation and/or transition to level walking. Results from Alcock et al. (77) show large differences in hip, knee and ankle sagittal angles during steady-state stair ascent and the transition from gait to stair ascent. Hence, both of the retrieved studies give information on stair transition but not on steady-state stair walking.

### 4.3. Effects of hip osteoarthritis on turning kinematics

In their study on 45° turns during walking, Tateuchi and colleagues (63) found reduced peak hip extension angles, similar to level walking. Additionally, peak hip flexion was also reduced. The step turn has been found to require more hip abduction than straight walking, while the spin turn requires greater hip adduction (78). HOA subjects showed decreased peak abduction or adduction during these tasks, respectively (63), but our meta-analysis did not yield these results for straight walking. Thus, analysis of turning while walking might be a beneficial extension of common gait analysis, especially as turning movements are encountered frequently during activities of daily living (79). However, further studies on turning are needed to confirm these results and also to expand the knowledge on transverse plane kinematics, which are also more demanding during turning than straight walking (78).

### 4.4. Limitations

Alongside the strengths of our study, there are also some limitations.

As stated before, a lot of the calculations were based on a limited number of studies and thus have to be interpreted with caution. Yet, the results might still be helpful to identify research gaps and support hypothesis formulation for future studies.

To have sufficient data for the meta-analysis, studies using different gait conditions (overground vs. treadmill) as well as different walking speeds and subject characteristics were included in the same analysis. Thus, the calculated SMD represents the average effect across a variety of study designs, measurement methods and subject populations. We therefore included prediction intervals in our analyses to provide a range for a potential HOA effect that might occur in individual study settings. Most of the significant results of our meta-analysis prediction intervals cross zero, meaning that in some settings no difference between HOA and CON subjects might be present. However, it has to be borne in mind that prediction intervals may be overly wide when they are calculated from a limited number of studies or from studies at a higher risk of bias (80).

Likewise, we included the *I*² statistic to estimate between-study heterogeneity. However, the *I*^2^ value can be imprecise and biased (81), especially in small meta-analyses such as the present one. Therefore, *I*^2^ estimates have to be interpreted with caution.

Lastly, we refrained from calculating a meta-regression to explore potential sources for between-study heterogeneity due to the small number of included studies (26).

### 4.5. Conclusion

In summary, this was the first review to synthesise data on lower body joint kinematics during locomotion movements in HOA subjects. A total of 47 reports from 35 individual studies were retrieved.

Most studies focused on gait, where kinematic differences were found in the ipsi- and contralateral hip and knee joints. While changes at the hip occurred in all 3 motion planes, changes in knee kinematics occurred mainly in the sagittal plane. Differences were found between subjects with mild or moderate HOA and those with severe HOA. Thus, motion analysis for HOA patients should not exclusively focus on the kinematics of the affected hip joint but also include analysis of adjacent and contralateral joints. Despite no significant results of the meta-analysis in ankle or pelvis kinematics, several indications exist for further analyses in this area. Additionally, 3 studies on stair walking and turning while walking were reviewed, and both of these movement tasks might be promising extensions to clinical movement analysis due to their elevated requirements on joint mobility.

Overall, large heterogeneity was observed across studies, so future studies have to further clarify the role of factors such as OA severity, laterality, age, gender, study design or movement execution in the analysis of lower limb joint kinematics.

## Data Availability

The original contributions presented in the study are included in the article/**Supplementary Material**, further inquiries can be directed to the corresponding author.
