# Supplementary material for: Effects of hip osteoarthritis on lower body joint kinematics during locomotion tasks: a systematic review and meta-analysis
Source: Front Sports Act Living. 2023 Nov 17;5:1197883. doi: 10.3389/fspor.2023.1197883 (PMC10690786; doi:10.3389/fspor.2023.1197883)
Supplement: Supplementary file 1 [file Datasheet1.pdf]

Supplementary Material

Effects of hip osteoarthritis on lower body joint kinematics during locomotion tasks: a systematic review and meta-analysis

Hannah Steingrebe*, Sina Spancken, Stefan Sell, Thorsten Stein

*** Correspondence:** Hannah Steingrebe: Hannah.steingrebe@kit.edu

Supplementary Table 1. Search terms used for literature search

Database	Search term	Filters	No. of results
Pubmed	((coxarthr*[Title/Abstract] OR ("degenerative joint"[Title/Abstract] AND hip[Title/Abstract]) OR (hip[Title/Abstract] AND osteoarthr*[Title/Abstract])) AND (kinematic*[Title/Abstract] OR angle*[Title/Abstract] OR "range of motion"[Title/Abstract] OR mobility[Title/Abstract] OR pattern[Title/Abstract] OR goniometric*[Title/Abstract] OR biomechanic*[Title/Abstract]) AND (gait[Title/Abstract] OR walk*[Title/Abstract] OR locomotion[Title/Abstract] OR ambulat*[Title/Abstract] OR stair*[Title/Abstract] OR movement[Title/Abstract]) NOT (fracture[Title] OR perthes[Title] OR amputee[Title] OR rheumat*[Title] OR arthroscopy[Title] OR Arthroplasty[Title]))	Human subjects	637
Web of science	TS=((coxarthr* OR (“degenerative joint” AND hip) OR (hip AND osteoarthr*)) AND (kinematic* OR angle* OR “range of motion” OR mobility OR pattern OR goniometric* OR biomechanic*) AND (gait OR walk* OR locomotion OR ambulat* OR stair*OR movement)) NOT TI=(fracture OR perthes OR amputee OR rheumat* OR arthroscopy OR Arthroplasty)		1370
Scopus	TITLE-ABS-KEY ((coxarthr* OR ("degenerative joint" AND hip) OR (hip AND osteoarthr*)) AND (kinematic* OR angle* OR "range of motion" OR mobility OR pattern OR goniometric* OR biomechanic*) AND (gait OR walk* OR locomotion OR ambulat* OR stair* OR movement)) AND NOT TITLE (fracture OR perthes OR amputee OR rheumat* OR arthroscopy OR Arthroplasty)		1678

(A)

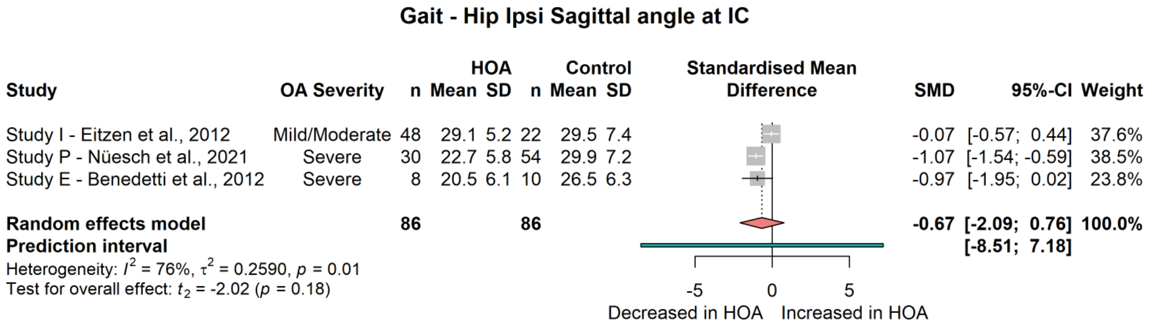

(B)

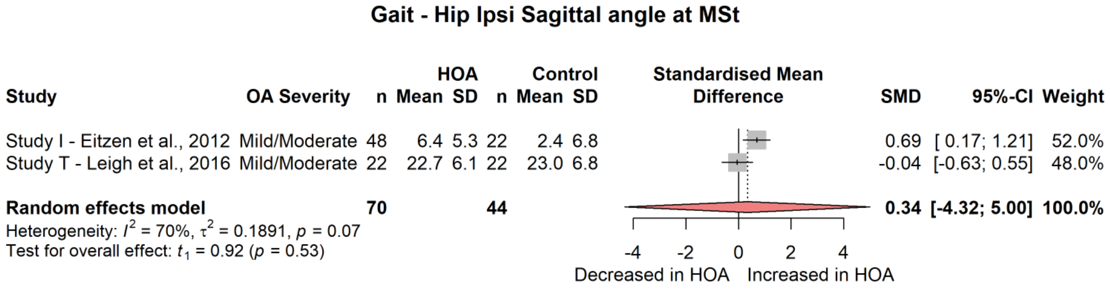

(C)

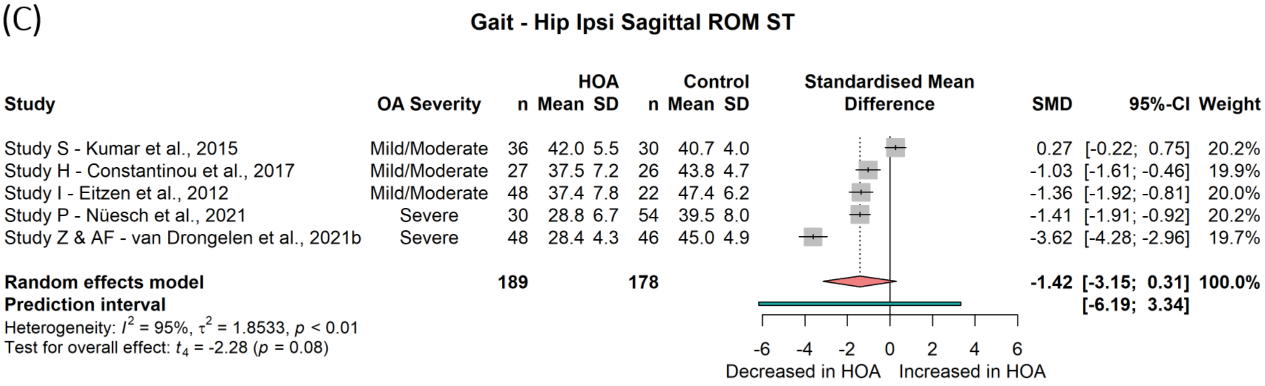

Supplementary Figure 2. Forest plot of standardised and pooled effect sizes (random-effects-model) with I² heterogeneity statistics for (A) ipsilateral hip sagittal angle at initial contact (IC), (B) ipsilateral hip sagittal angle at midstance (MSt), (C) ipsilateral hip sagittal range of motion (ROM) across stance phase; during gait.

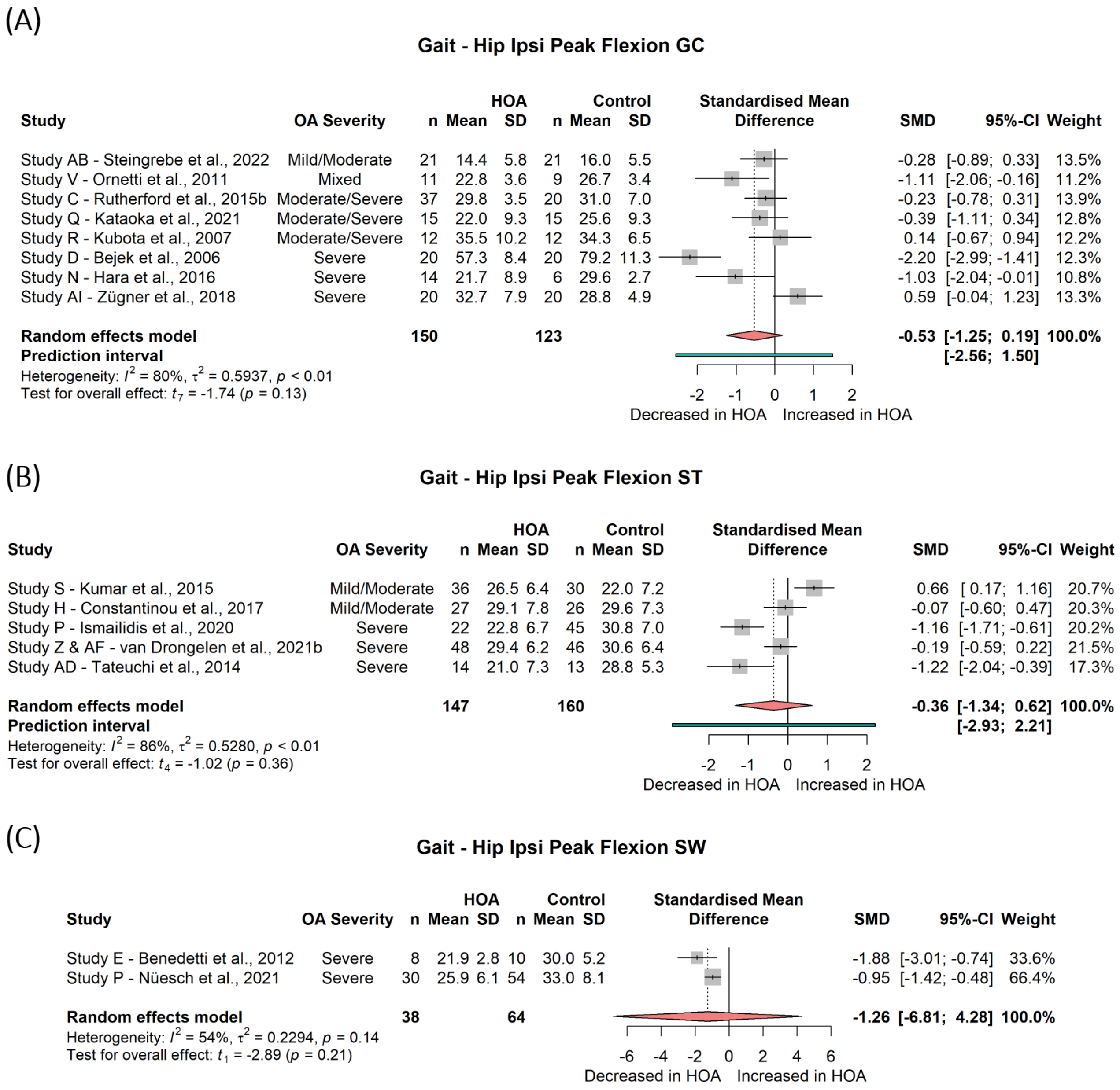

Supplementary Figure 2. Forest plot of standardised and pooled effect sizes (random-effects-model) with I² heterogeneity statistics for (A) ipsilateral peak hip flexion during gait cycle (GC), (B) ipsilateral peak hip flexion during stance phase (ST), (C) ipsilateral peak hip flexion during swing phase (SW); during gait.

(A)

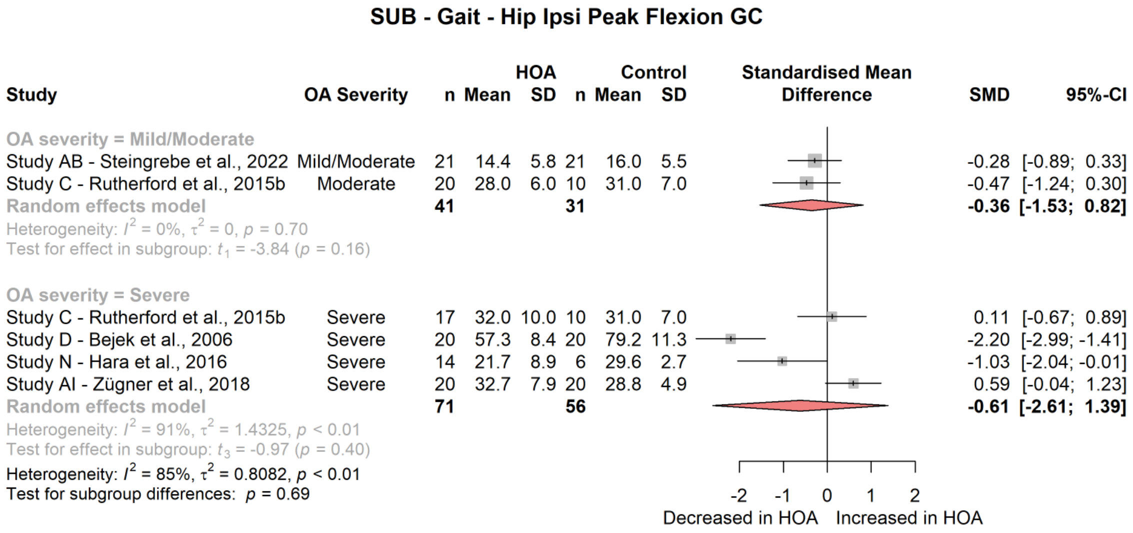

(B)

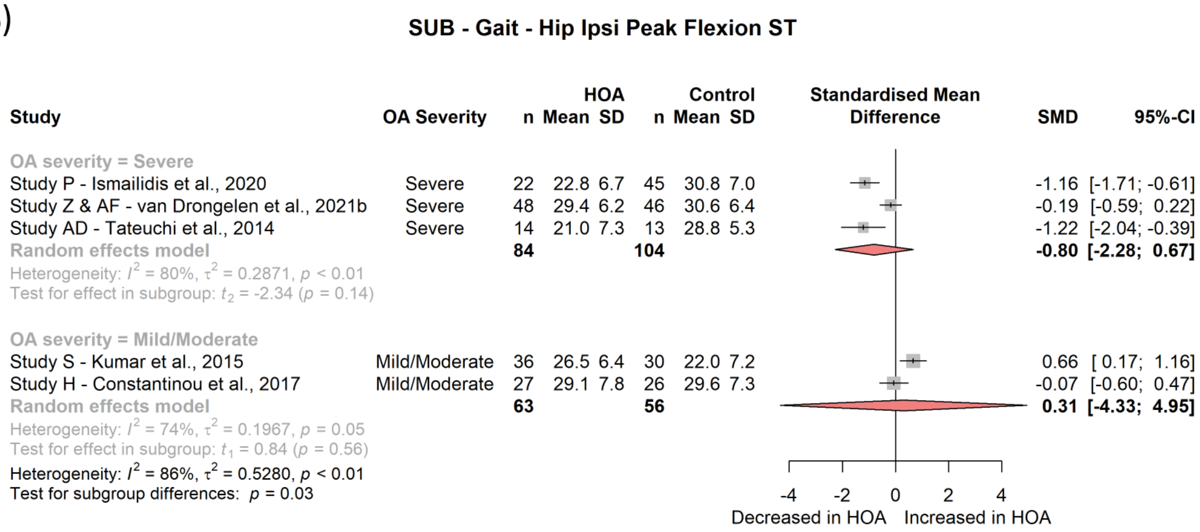

Supplementary Figure 3. Forest plot of standardised and pooled effect sizes (random-effects-model) with I^2 heterogeneity statistics for subgroup analyses on (A) ipsilateral peak hip flexion during gait cycle (GC), (B) ipsilateral peak hip flexion during stance phase (ST); during gait.

(A)

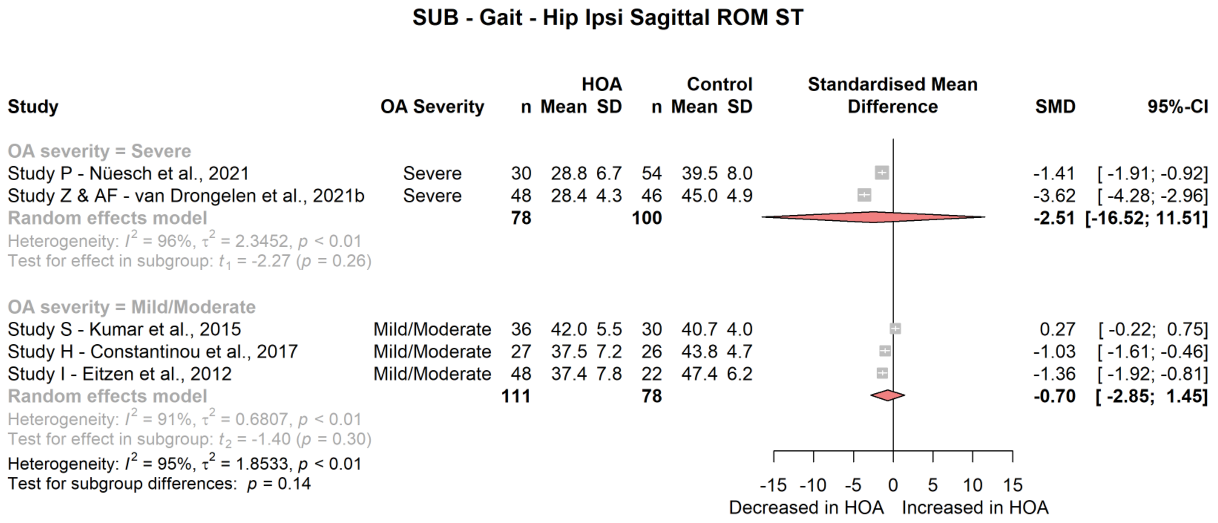

(B)

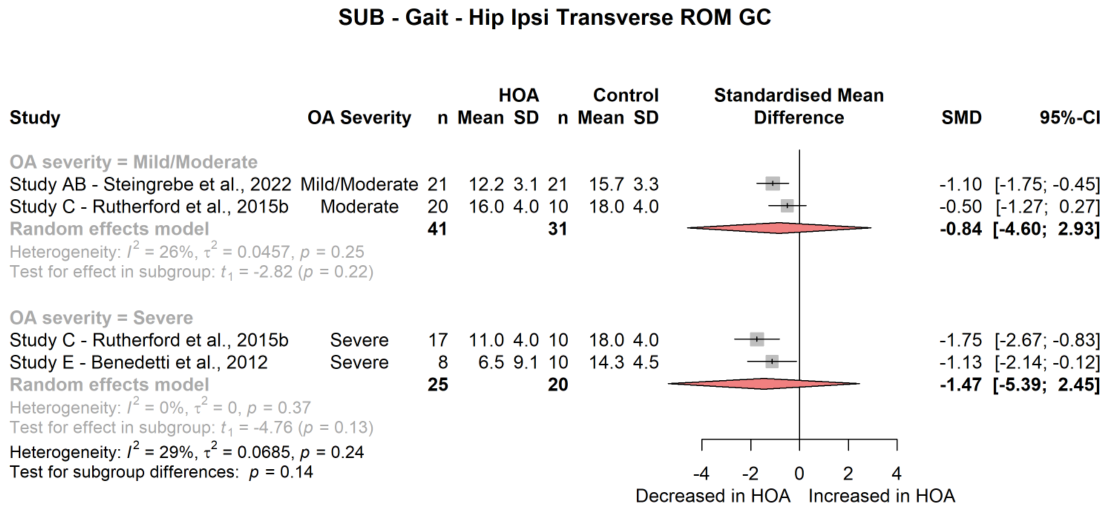

Supplementary Figure 4. Forest plot of standardised and pooled effect sizes (random-effects-model) with I^2 heterogeneity statistics for subgroup analyses on (A) ipsilateral hip sagittal range of motion (ROM) across stance phase (ST), (B) ipsilateral hip transverse ROM across gait cycle (GC); during gait

(A)

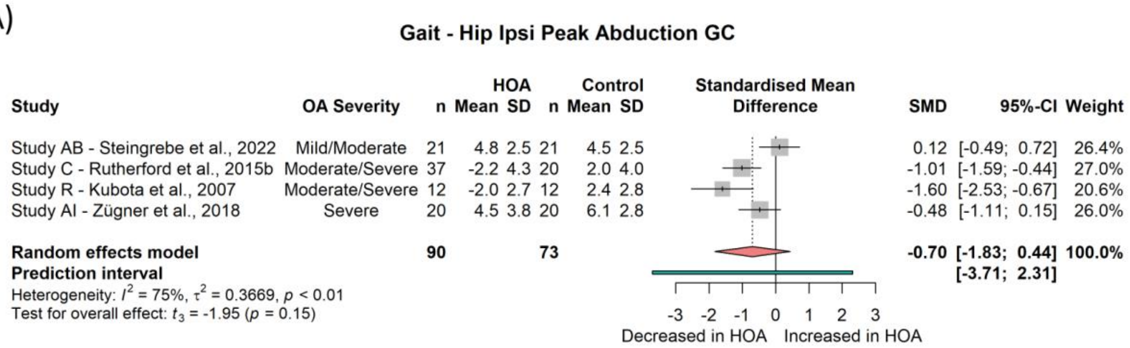
Gait - Hip Ipsi Peak Abduction GC
Study | OA Severity | HOA n Mean SD | Control n Mean SD | Standardised Mean Difference | SMD | 95%-CI | Weight
Study AB - Steingrebe et al., 2022 | Mild/Moderate | 21 4.8 2.5 | 21 4.5 2.5 | 0.12 | [-0.49; 0.72] | 26.4%
Study C - Rutherford et al., 2015b | Moderate/Severe | 37 -2.2 4.3 | 20 2.0 4.0 | -1.01 | [-1.59; -0.44] | 27.0%
Study R - Kubota et al., 2007 | Moderate/Severe | 12 -2.0 2.7 | 12 2.4 2.8 | -1.60 | [-2.53; -0.67] | 20.6%
Study AI - Zügner et al., 2018 | Severe | 20 4.5 3.8 | 20 6.1 2.8 | -0.48 | [-1.11; 0.15] | 26.0%
Random effects model | 90 | 73 | -0.70 | [-1.83; 0.44] | 100.0%
Prediction interval | [-3.71; 2.31]
Heterogeneity: I² = 75%, τ² = 0.3669, p < 0.01
Test for overall effect: t₃ = -1.95 (p = 0.15)
-3 -2 -1 0 1 2 3
Decreased in HOA Increased in HOA

(B)

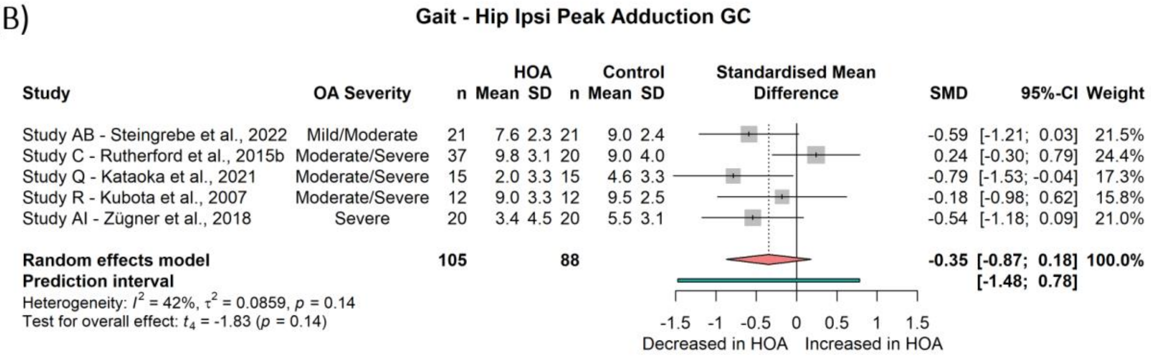
Gait - Hip Ipsi Peak Adduction GC
Study | OA Severity | HOA n Mean SD | Control n Mean SD | Standardised Mean Difference | SMD | 95%-CI | Weight
Study AB - Steingrebe et al., 2022 | Mild/Moderate | 21 7.6 2.3 | 21 9.0 2.4 | -0.59 | [-1.21; 0.03] | 21.5%
Study C - Rutherford et al., 2015b | Moderate/Severe | 37 9.8 3.1 | 20 9.0 4.0 | 0.24 | [-0.30; 0.79] | 24.4%
Study Q - Kataoka et al., 2021 | Moderate/Severe | 15 2.0 3.3 | 15 4.6 3.3 | -0.79 | [-1.53; -0.04] | 17.3%
Study R - Kubota et al., 2007 | Moderate/Severe | 12 9.0 3.3 | 12 9.5 2.5 | -0.18 | [-0.98; 0.62] | 15.8%
Study AI - Zügner et al., 2018 | Severe | 20 3.4 4.5 | 20 5.5 3.1 | -0.54 | [-1.18; 0.09] | 21.0%
Random effects model | 105 | 88 | -0.35 | [-0.87; 0.18] | 100.0%
Prediction interval | [-1.48; 0.78]
Heterogeneity: I² = 42%, τ² = 0.0859, p = 0.14
Test for overall effect: t₄ = -1.83 (p = 0.14)
-1.5 -1 -0.5 0 0.5 1 1.5
Decreased in HOA Increased in HOA

(C)

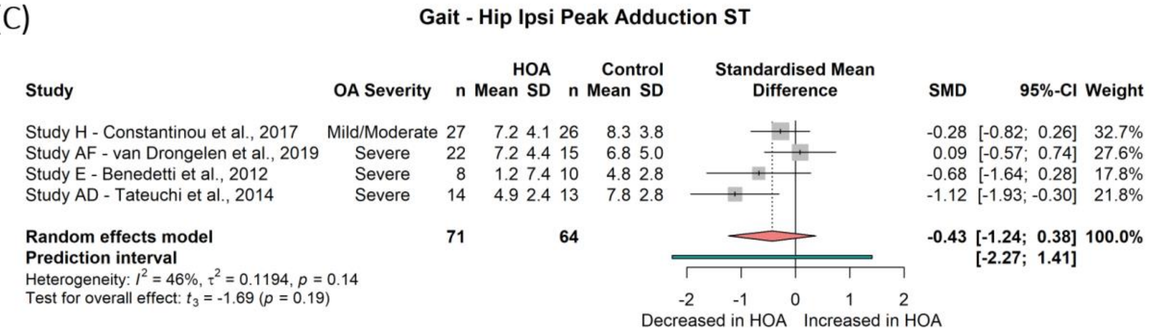
Gait - Hip Ipsi Peak Adduction ST
Study | OA Severity | HOA n Mean SD | Control n Mean SD | Standardised Mean Difference | SMD | 95%-CI | Weight
Study H - Constantinou et al., 2017 | Mild/Moderate | 27 7.2 4.1 | 26 8.3 3.8 | -0.28 | [-0.82; 0.26] | 32.7%
Study AF - van Drongelen et al., 2019 | Severe | 22 7.2 4.4 | 15 6.8 5.0 | 0.09 | [-0.57; 0.74] | 27.6%
Study E - Benedetti et al., 2012 | Severe | 8 1.2 7.4 | 10 4.8 2.8 | -0.68 | [-1.64; 0.28] | 17.8%
Study AD - Tateuchi et al., 2014 | Severe | 14 4.9 2.4 | 13 7.8 2.8 | -1.12 | [-1.93; -0.30] | 21.8%
Random effects model | 71 | 64 | -0.43 | [-1.24; 0.38] | 100.0%
Prediction interval | [-2.27; 1.41]
Heterogeneity: I² = 46%, τ² = 0.1194, p = 0.14
Test for overall effect: t₃ = -1.69 (p = 0.19)
-2 -1 0 1 2
Decreased in HOA Increased in HOA

(D)

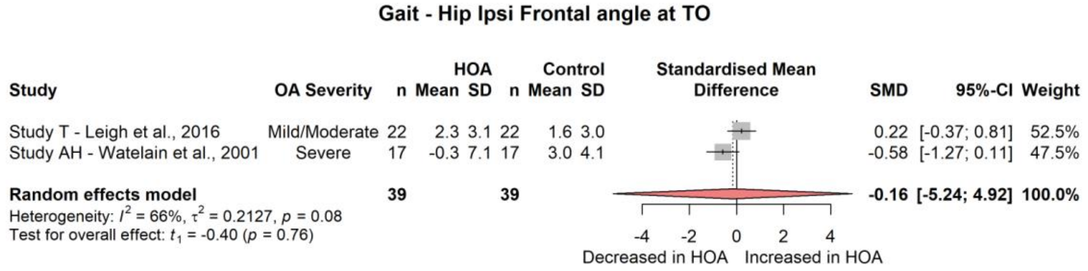
Gait - Hip Ipsi Frontal angle at TO
Study | OA Severity | HOA n Mean SD | Control n Mean SD | Standardised Mean Difference | SMD | 95%-CI | Weight
Study T - Leigh et al., 2016 | Mild/Moderate | 22 2.3 3.1 | 22 1.6 3.0 | 0.22 | [-0.37; 0.81] | 52.5%
Study AH - Watelain et al., 2001 | Severe | 17 -0.3 7.1 | 17 3.0 4.1 | -0.58 | [-1.27; 0.11] | 47.5%
Random effects model | 39 | 39 | -0.16 | [-5.24; 4.92] | 100.0%
Heterogeneity: I² = 66%, τ² = 0.2127, p = 0.08
Test for overall effect: t₁ = -0.40 (p = 0.76)
-4 -2 0 2 4
Decreased in HOA Increased in HOA

(E)

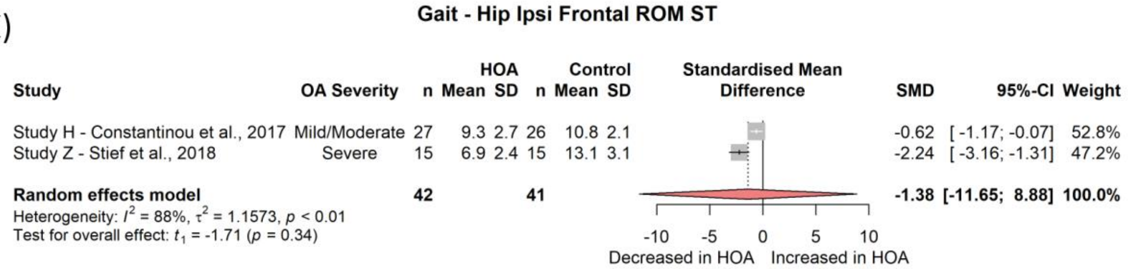
Gait - Hip Ipsi Frontal ROM ST
Study | OA Severity | HOA n Mean SD | Control n Mean SD | Standardised Mean Difference | SMD | 95%-CI | Weight
Study H - Constantinou et al., 2017 | Mild/Moderate | 27 9.3 2.7 | 26 10.8 2.1 | -0.62 | [-1.17; -0.07] | 52.8%
Study Z - Stief et al., 2018 | Severe | 15 6.9 2.4 | 15 13.1 3.1 | -2.24 | [-3.16; -1.31] | 47.2%
Random effects model | 42 | 41 | -1.38 | [-11.65; 8.88] | 100.0%
Heterogeneity: I² = 88%, τ² = 1.1573, p < 0.01
Test for overall effect: t₁ = -1.71 (p = 0.34)
-10 -5 0 5 10
Decreased in HOA Increased in HOA

Supplementary Figure 5. Forest plot of standardised and pooled effect sizes (random-effects-model) with I^2 heterogeneity statistics for (A) ipsilateral peak hip abduction during gait cycle (GC), (B) ipsilateral peak hip adduction during GC, (C) ipsilateral peak hip adduction during stance phase (ST), (D) ipsilateral frontal hip angle at toe-off (TO), (E) ipsilateral hip frontal range of motion (ROM) across ST; during gait.

(A)

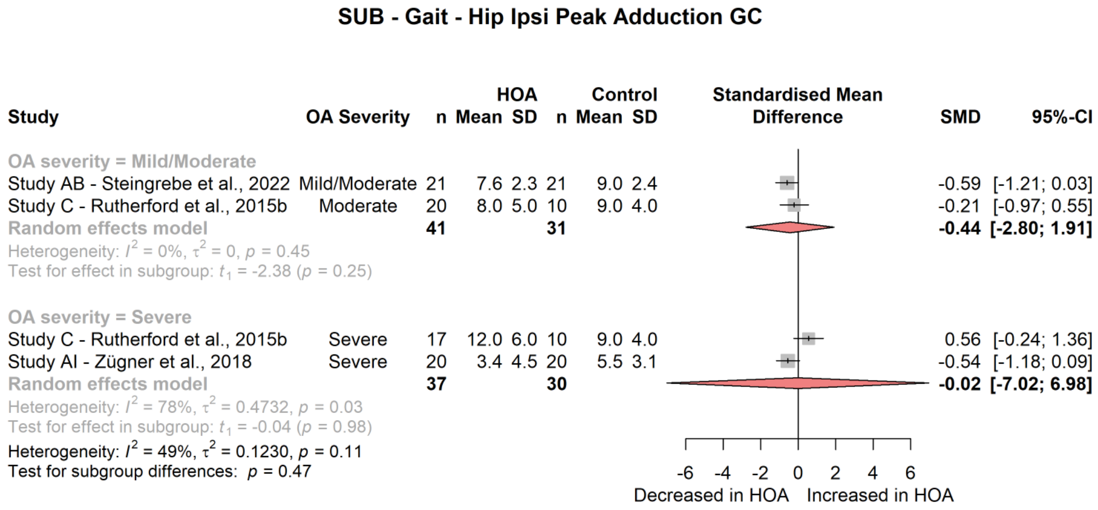

(B)

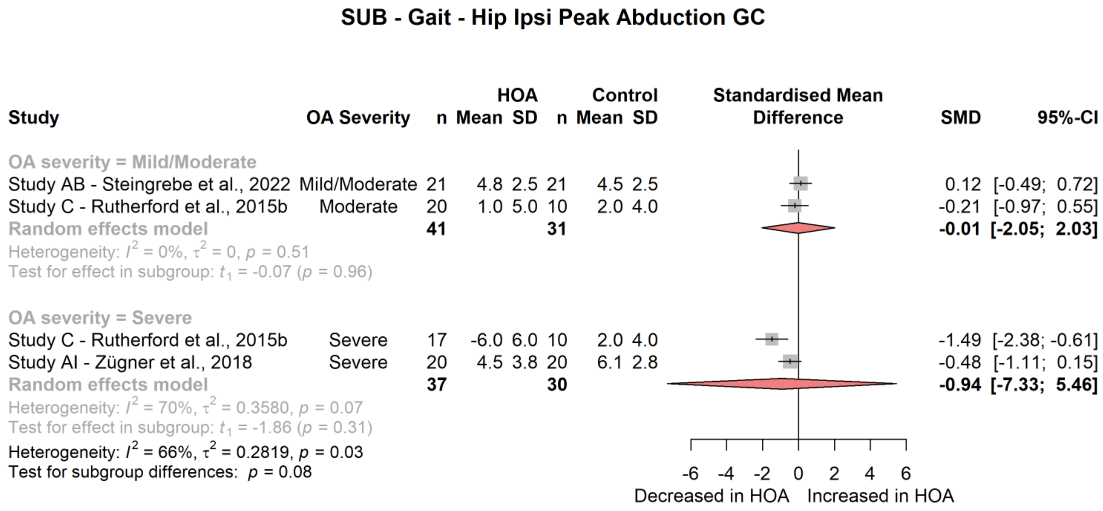

Supplementary Figure 6. Forest plot of standardised and pooled effect sizes (random-effects-model) with I^2 heterogeneity statistics for subgroup analyses on (A) ipsilateral hip peak adduction during gait cycle (GC), (B) ipsilateral hip peak abduction during GC; during gait

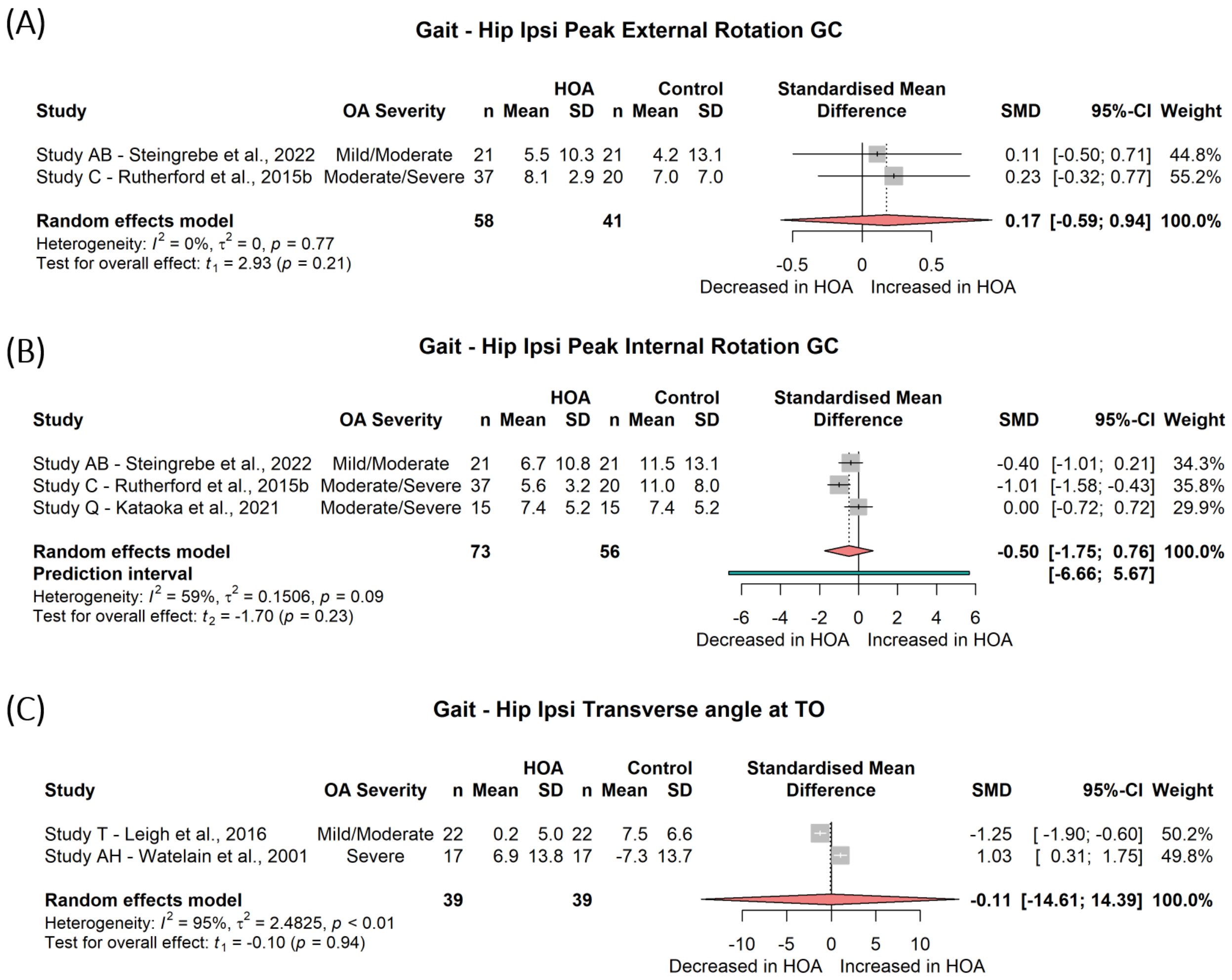

Supplementary Figure 7. Forest plot of standardised and pooled effect sizes (random-effects-model) with I² heterogeneity statistics for (A) ipsilateral hip peak external rotation during gait cycle (GC), (B) ipsilateral hip peak internal rotation during GC, (C) ipsilateral hip transverse angle at toe-off (TO); during gait.

(A)

Gait - Hip Contra Peak Extension GC

Study	OA Severity	HOA n	HOA Mean	HOA SD	Control n	Control Mean	Control SD	SMD	95%-CI	Weight
Study V - Ornetti et al., 2011	Mixed	11	9.0	6.5	9	13.3	3.4	-0.80	[-1.72; 0.12]	45.9%
Study D - Bejek et al., 2006	Severe	20	48.1	10.8	20	43.2	13.7	0.40	[-0.23; 1.02]	54.1%
**Random effects model**		**31**			**29**			**-0.15**	**[-7.76; 7.45]**	**100.0%**

Heterogeneity: $I^2 = 78\%$, $\tau^2 = 0.5602$, $p = 0.03$
Test for overall effect: $t_1 = -0.26$ ($p = 0.84$)

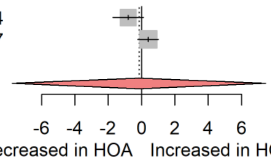

(B)

Gait - Hip Contra Peak Flexion GC

Study	OA Severity	HOA n	HOA Mean	HOA SD	Control n	Control Mean	Control SD	SMD	95%-CI	Weight
Study V - Ornetti et al., 2011	Mixed	11	23.9	6.6	9	26.7	3.4	-0.52	[-1.41; 0.38]	34.1%
Study D - Bejek et al., 2006	Severe	20	70.3	3.7	20	80.7	18.1	-0.80	[-1.44; -0.15]	65.9%
**Random effects model**		**31**			**29**			**-0.70**	**[-2.38; 0.98]**	**100.0%**

Heterogeneity: $I^2 = 0\%$, $\tau^2 = 0$, $p = 0.62$
Test for overall effect: $t_1 = -5.30$ ($p = 0.12$)

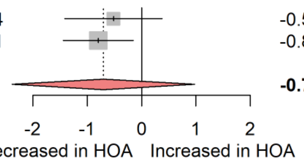

(C)

Gait - Hip Contra Peak Flexion ST

Study	OA Severity	HOA n	HOA Mean	HOA SD	Control n	Control Mean	Control SD	SMD	95%-CI	Weight
Study Z - Stief et al., 2018	Severe	15	35.5	7.7	15	32.4	6.7	0.43	[-0.30; 1.15]	23.8%
Study AF - van Drongelen et al., 2019	Severe	22	37.9	10.9	15	33.9	5.7	0.44	[-0.23; 1.10]	28.3%
Study P - Ismailidis et al., 2020	Severe	22	30.9	8.5	45	30.8	7.0	0.01	[-0.50; 0.52]	48.0%
**Random effects model**		**59**			**75**			**0.23**	**[-0.41; 0.87]**	**100.0%**
**Prediction interval**									**[-2.06; 2.52]**	

Heterogeneity: $I^2 = 0\%$, $\tau^2 = 0$, $p = 0.51$
Test for overall effect: $t_2 = 1.56$ ($p = 0.26$)

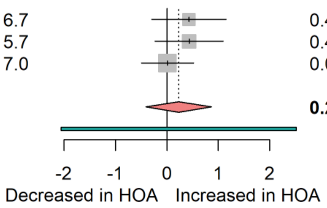

(D)

Gait - Hip Contra Sagittal ROM GC

Study	OA Severity	HOA n	HOA Mean	HOA SD	Control n	Control Mean	Control SD	SMD	95%-CI	Weight
Study V - Ornetti et al., 2011	Mixed	11	32.9	4.2	9	40.0	3.2	-1.87	[-2.95; -0.80]	22.9%
Study D - Bejek et al., 2006	Severe	20	22.2	8.1	20	37.3	8.9	-1.77	[-2.51; -1.04]	25.7%
Study O - Hurwitz et al., 1997	Severe	19	31.0	6.0	19	29.0	6.0	0.33	[-0.31; 0.97]	26.4%
Study X - Porta et al., 2021	Severe	11	43.0	8.4	11	42.8	4.6	0.03	[-0.81; 0.87]	24.9%
**Random effects model**		**61**			**59**			**-0.79**	**[-2.65; 1.06]**	**100.0%**
**Prediction interval**									**[-6.11; 4.53]**	

Heterogeneity: $I^2 = 88\%$, $\tau^2 = 1.1858$, $p < 0.01$
Test for overall effect: $t_3 = -1.36$ ($p = 0.27$)

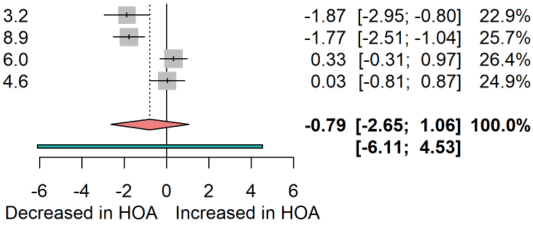

(E)

Gait - Hip Contra Sagittal ROM ST

Study	OA Severity	HOA n	HOA Mean	HOA SD	Control n	Control Mean	Control SD	SMD	95%-CI	Weight
Study Z - Stief et al., 2018	Severe	15	42.5	6.4	15	43.7	3.7	-0.23	[-0.95; 0.49]	21.1%
Study AF - van Drongelen et al., 2019	Severe	22	43.1	9.4	15	43.7	3.7	-0.08	[-0.73; 0.58]	25.3%
Study P - Nüesch et al., 2021	Severe	30	36.7	10.2	54	39.8	5.1	-0.42	[-0.87; 0.03]	53.6%
**Random effects model**		**67**			**84**			**-0.30**	**[-0.74; 0.15]**	**100.0%**
**Prediction interval**									**[-2.44; 1.85]**	

Heterogeneity: $I^2 = 0\%$, $\tau^2 = 0$, $p = 0.68$
Test for overall effect: $t_2 = -2.84$ ($p = 0.10$)

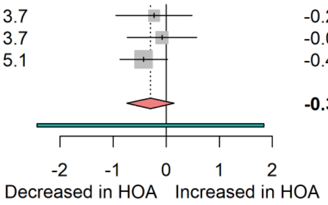

Supplementary Figure 8. Forest plot of standardised and pooled effect sizes (random-effects-model) with I^2 heterogeneity statistics for (A) contralateral peak hip extension during gait cycle (GC), (B) contralateral peak hip flexion during GC, (C) contralateral peak hip flexion during stance phase (ST), (D) contralateral hip sagittal range of motion (ROM) across GC, (E) contralateral hip sagittal ROM ST; during gait.

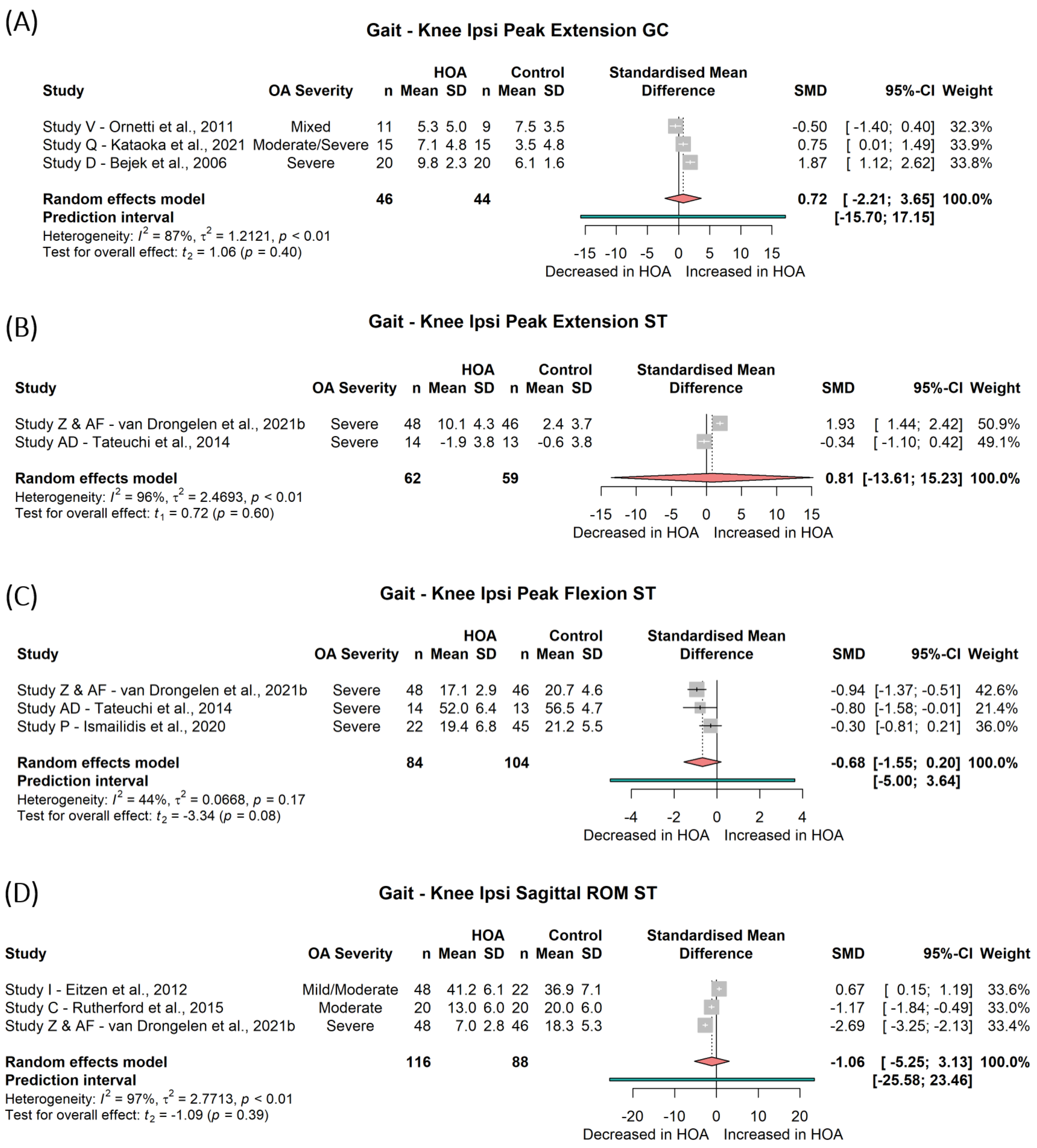

Supplementary Figure 9. Forest plot of standardised and pooled effect sizes (random-effects-model) with I² heterogeneity statistics for (A) ipsilateral peak knee extension during gait cycle (GC), (B) ipsilateral peak knee extension during stance phase (ST), (C) ipsilateral peak knee flexion ST, (D) ipsilateral knee sagittal range of motion (ROM) across ST; during gait.

(A)

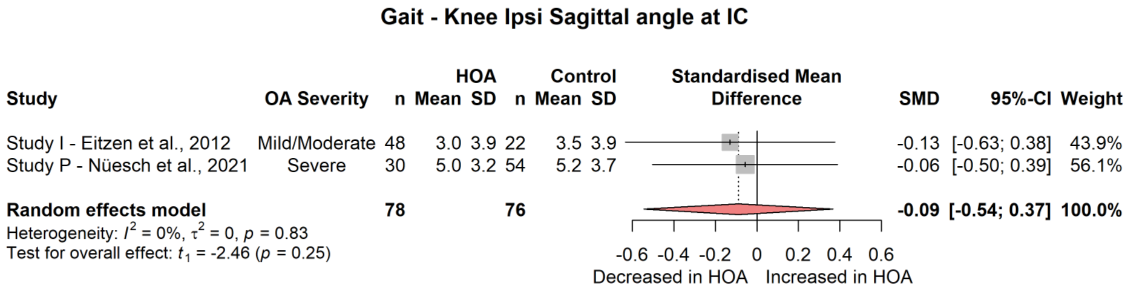

(B)

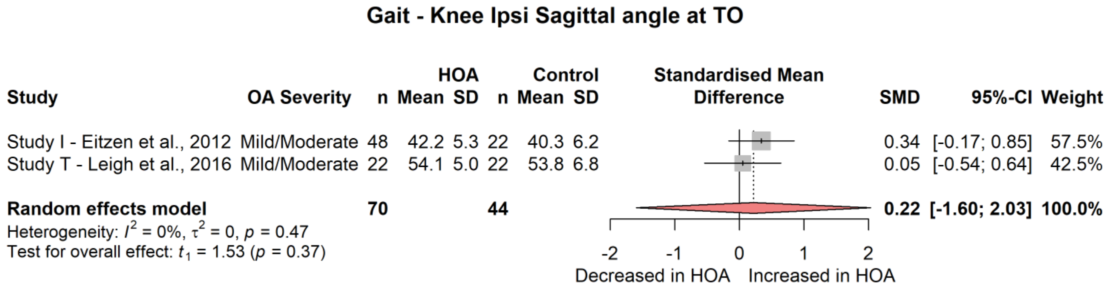

(C)

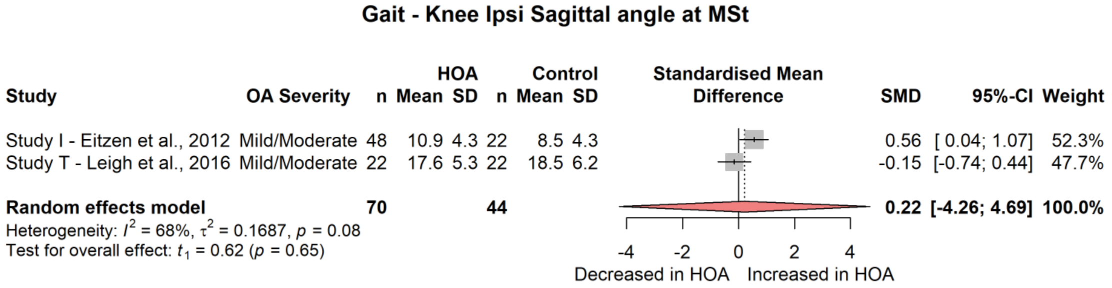

(D)

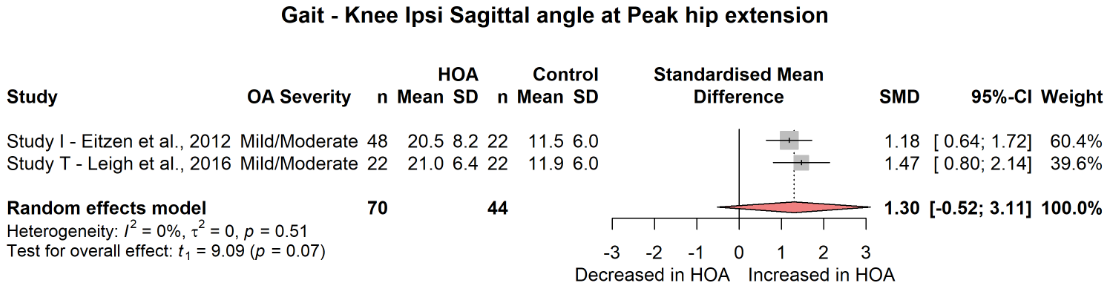

Supplementary Figure 10. Forest plot of standardised and pooled effect sizes (random-effects-model) with I² heterogeneity statistics for (A) ipsilateral knee sagittal angle at initial contact (IC), (B) ipsilateral knee sagittal angle at toe-off (TO), (C) ipsilateral knee sagittal angle at midstance (MSt), (D) ipsilateral knee sagittal angle at peak hip extension; during gait.

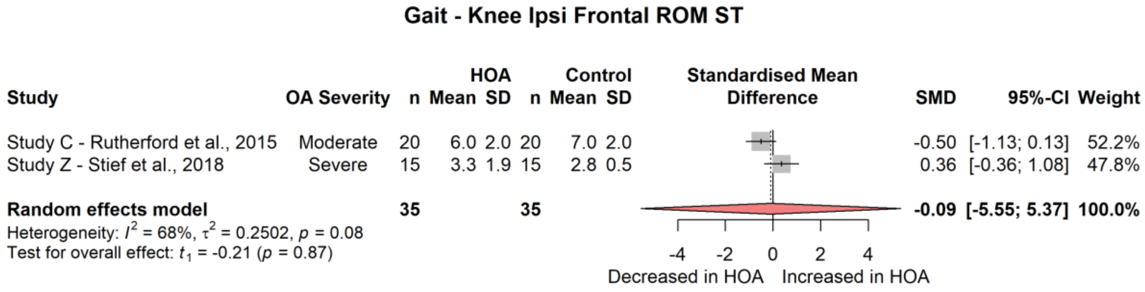

Supplementary Figure 11. Forest plot of standardised and pooled effect sizes (random-effects-model) with I^2 heterogeneity statistics for ipsilateral frontal knee range of motion (ROM) across stance phase (ST) during gait.

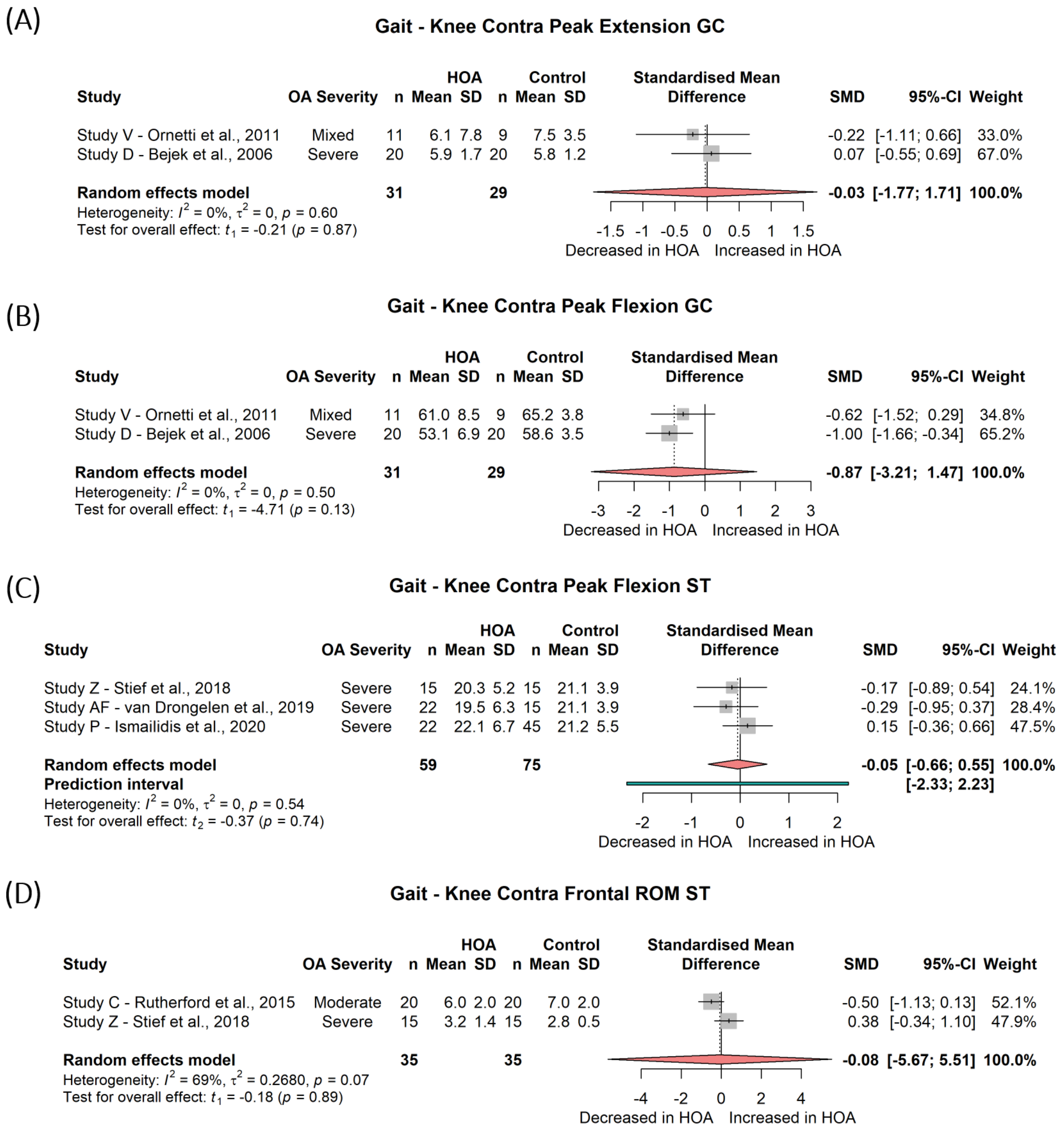

Supplementary Figure 12. Forest plot of standardised and pooled effect sizes (random-effects-model) with I² heterogeneity statistics for (A) contralateral peak knee extension during gait cycle (GC), (B) contralateral peak knee flexion during GC, (C) contralateral peak knee flexion during stance phase (ST), (D) contralateral knee frontal range of motion (ROM) across ST; during gait.

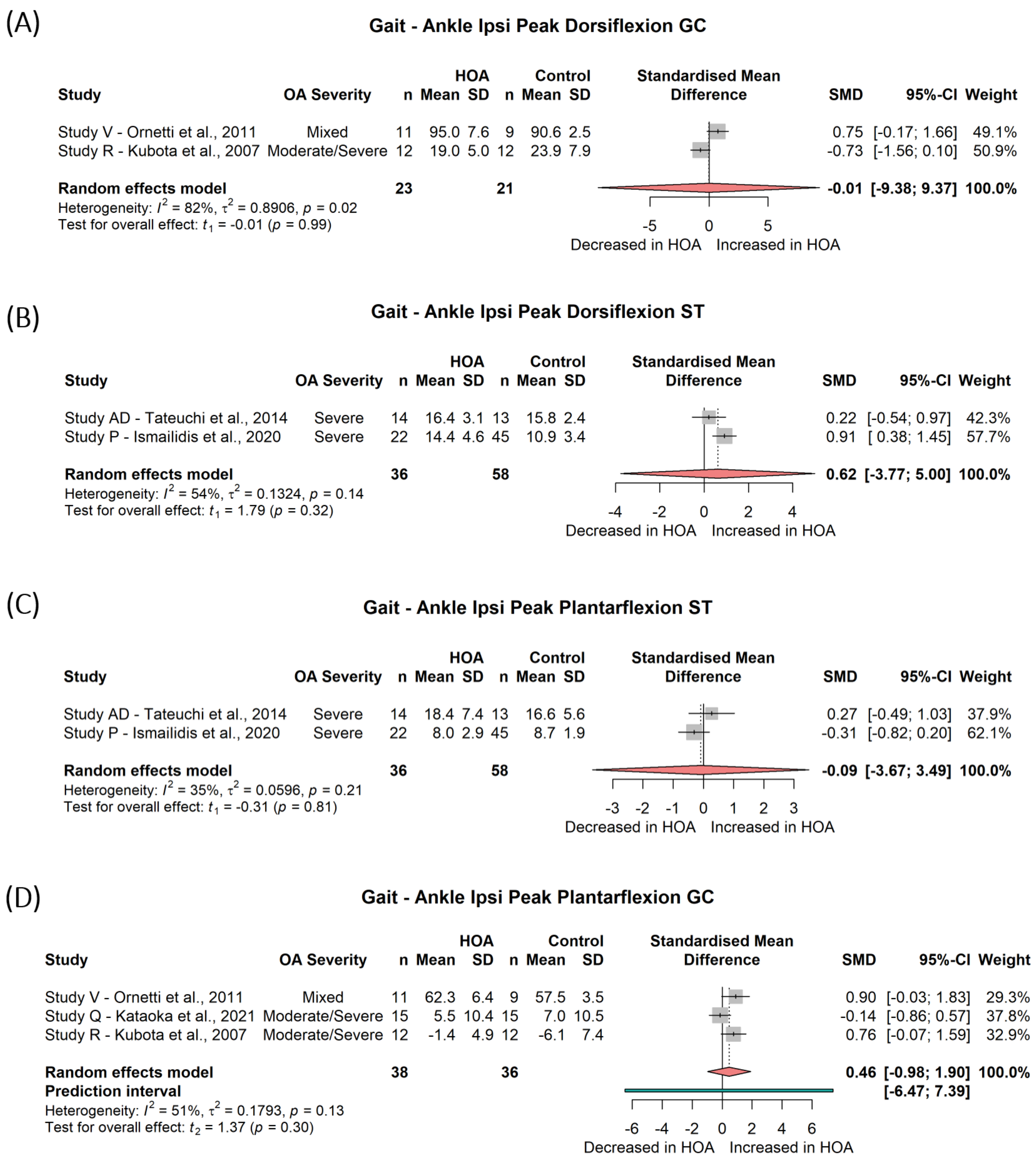

Supplementary Figure 13. Forest plot of standardised and pooled effect sizes (random-effects-model) with I² heterogeneity statistics for (A) ipsilateral peak ankle dorsiflexion during gait cycle (GC), (B) ipsilateral peak ankle dorsiflexion during stance phase (ST), (C) ipsilateral peak ankle plantarflexion ST, (D) ipsilateral peak ankle plantarflexion GC; during gait.

(A)

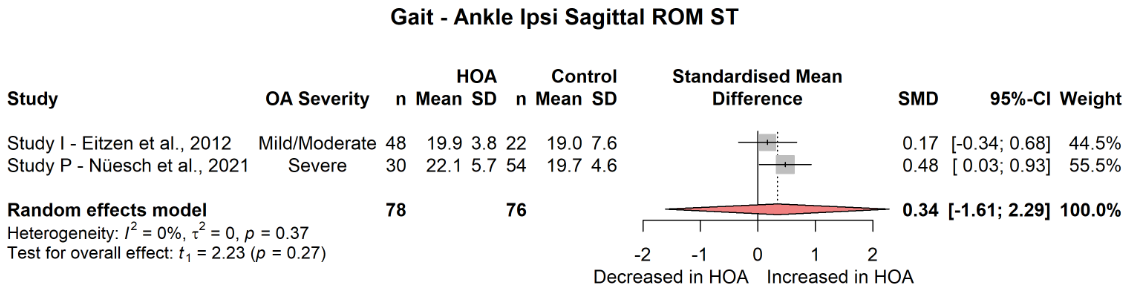

(B)

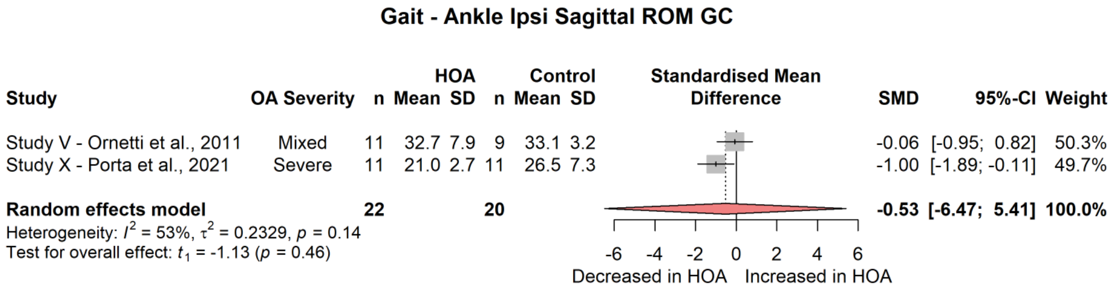

(C)

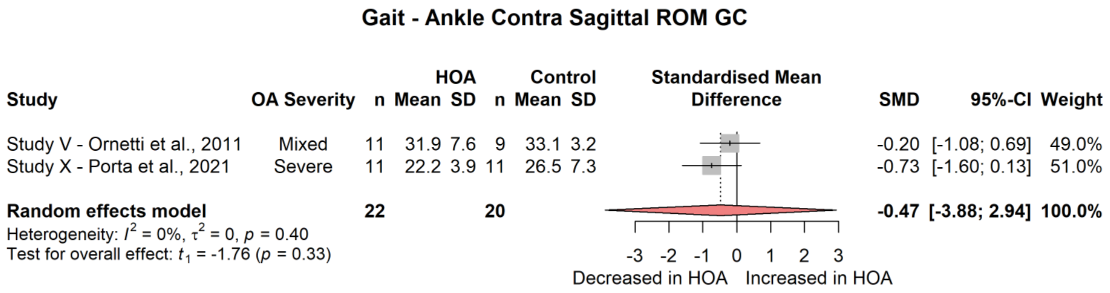

Supplementary Figure 14. Forest plot of standardised and pooled effect sizes (random-effects-model) with I^2 heterogeneity statistics for (A) ipsilateral ankle sagittal range of motion (ROM) across stance phase (ST), (B) ipsilateral ankle sagittal ROM across gait cycle (GC), (C) contralateral ankle sagittal ROM across GC; during gait.

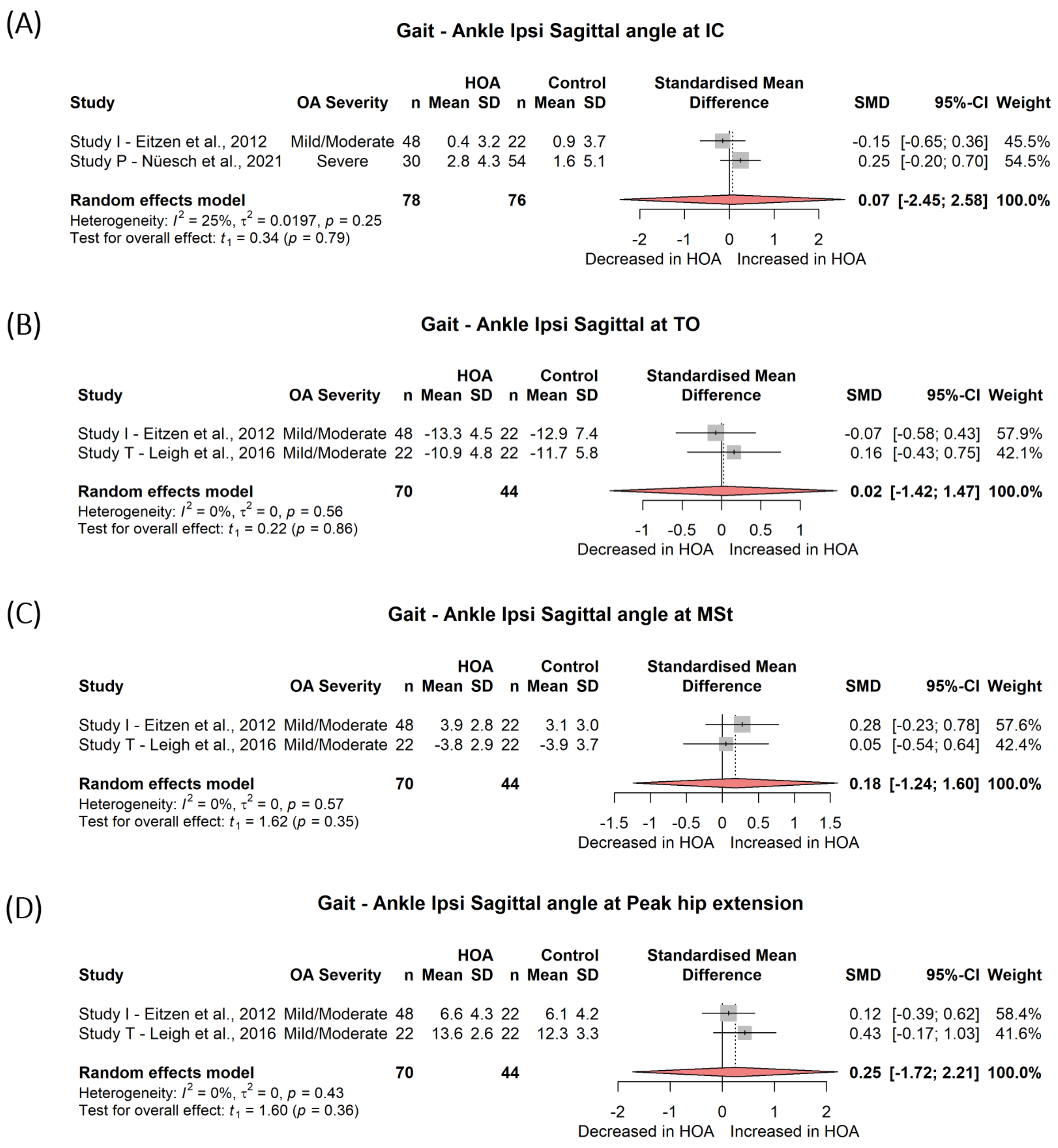

Supplementary Figure 15. Forest plot of standardised and pooled effect sizes (random-effects-model) with I² heterogeneity statistics for (A) ipsilateral ankle sagittal angle at initial contact (IC), (B) ipsilateral ankle sagittal angle at toe-off (TO), (C) ipsilateral ankle sagittal angle at midstance (MSt), (D) ipsilateral ankle sagittal angle at peak hip extension; during gait.

(A)

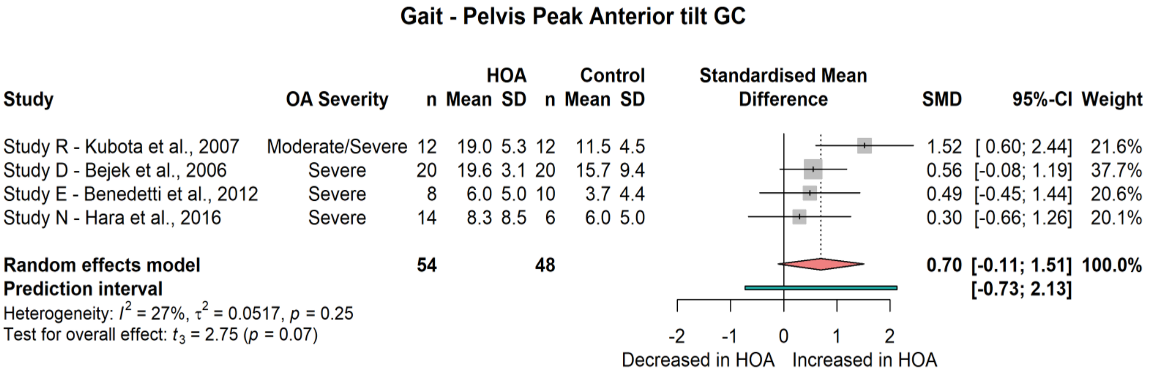
Gait - Pelvis Peak Anterior tilt GC
Study | OA Severity | HOA n Mean SD | Control n Mean SD | Standardised Mean Difference | SMD | 95%-CI | Weight
Study R - Kubota et al., 2007 Moderate/Severe 12 19.0 5.3 12 11.5 4.5 1.52 [0.60; 2.44] 21.6%
Study D - Bejek et al., 2006 Severe 20 19.6 3.1 20 15.7 9.4 0.56 [-0.08; 1.19] 37.7%
Study E - Benedetti et al., 2012 Severe 8 6.0 5.0 10 3.7 4.4 0.49 [-0.45; 1.44] 20.6%
Study N - Hara et al., 2016 Severe 14 8.3 8.5 6 6.0 5.0 0.30 [-0.66; 1.26] 20.1%
Random effects model 54 48 0.70 [-0.11; 1.51] 100.0%
Prediction interval [-0.73; 2.13]
Heterogeneity: I^2 = 27%, τ^2 = 0.0517, p = 0.25
Test for overall effect: t_3 = 2.75 (p = 0.07)
-2 -1 0 1 2
Decreased in HOA Increased in HOA

(B)

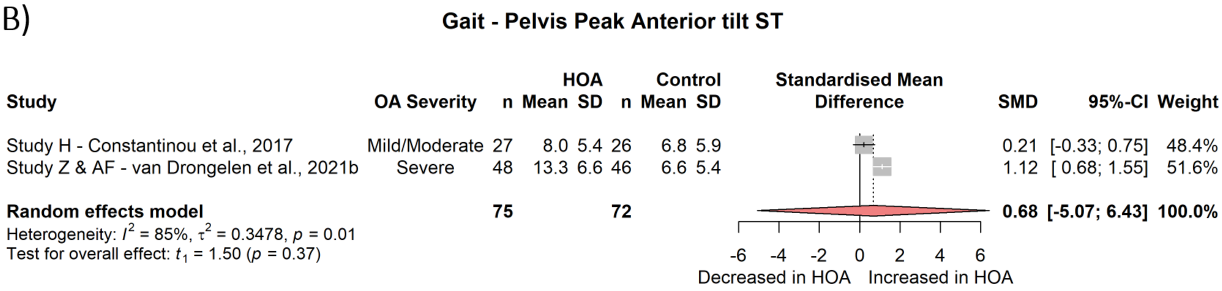
Gait - Pelvis Peak Anterior tilt ST
Study | OA Severity | HOA n Mean SD | Control n Mean SD | Standardised Mean Difference | SMD | 95%-CI | Weight
Study H - Constantinou et al., 2017 Mild/Moderate 27 8.0 5.4 26 6.8 5.9 0.21 [-0.33; 0.75] 48.4%
Study Z & AF - van Drongelen et al., 2021b Severe 48 13.3 6.6 46 6.6 5.4 1.12 [0.68; 1.55] 51.6%
Random effects model 75 72 0.68 [-5.07; 6.43] 100.0%
Heterogeneity: I^2 = 85%, τ^2 = 0.3478, p = 0.01
Test for overall effect: t_1 = 1.50 (p = 0.37)
-6 -4 -2 0 2 4 6
Decreased in HOA Increased in HOA

(C)

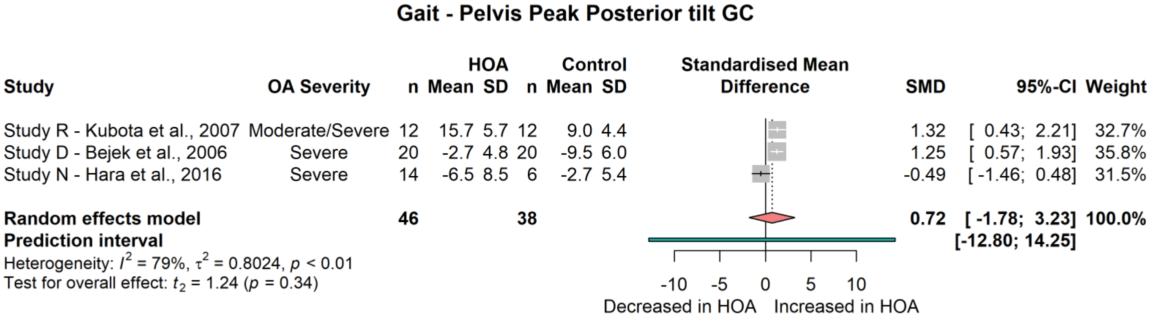
Gait - Pelvis Peak Posterior tilt GC
Study | OA Severity | HOA n Mean SD | Control n Mean SD | Standardised Mean Difference | SMD | 95%-CI | Weight
Study R - Kubota et al., 2007 Moderate/Severe 12 15.7 5.7 12 9.0 4.4 1.32 [0.43; 2.21] 32.7%
Study D - Bejek et al., 2006 Severe 20 -2.7 4.8 20 -9.5 6.0 1.25 [0.57; 1.93] 35.8%
Study N - Hara et al., 2016 Severe 14 -6.5 8.5 6 -2.7 5.4 -0.49 [-1.46; 0.48] 31.5%
Random effects model 46 38 0.72 [-1.78; 3.23] 100.0%
Prediction interval [-12.80; 14.25]
Heterogeneity: I^2 = 79%, τ^2 = 0.8024, p < 0.01
Test for overall effect: t_2 = 1.24 (p = 0.34)
-10 -5 0 5 10
Decreased in HOA Increased in HOA

(D)

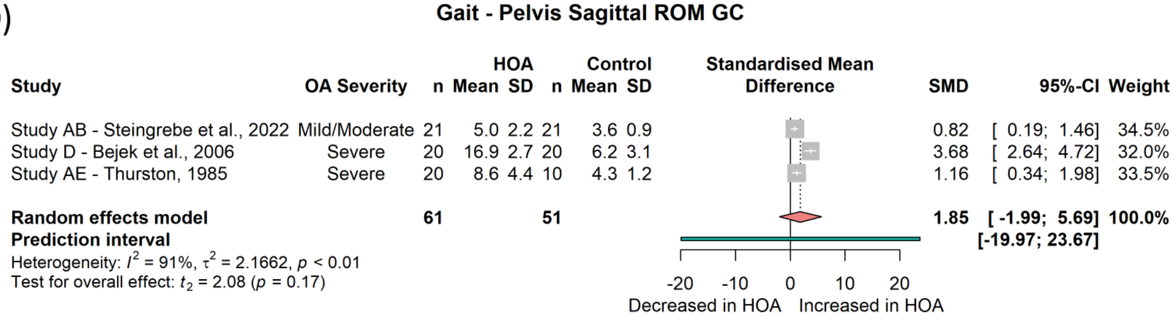
Gait - Pelvis Sagittal ROM GC
Study | OA Severity | HOA n Mean SD | Control n Mean SD | Standardised Mean Difference | SMD | 95%-CI | Weight
Study AB - Steingrebe et al., 2022 Mild/Moderate 21 5.0 2.2 21 3.6 0.9 0.82 [0.19; 1.46] 34.5%
Study D - Bejek et al., 2006 Severe 20 16.9 2.7 20 6.2 3.1 3.68 [2.64; 4.72] 32.0%
Study AE - Thurston, 1985 Severe 20 8.6 4.4 10 4.3 1.2 1.16 [0.34; 1.98] 33.5%
Random effects model 61 51 1.85 [-1.99; 5.69] 100.0%
Prediction interval [-19.97; 23.67]
Heterogeneity: I^2 = 91%, τ^2 = 2.1662, p < 0.01
Test for overall effect: t_2 = 2.08 (p = 0.17)
-20 -10 0 10 20
Decreased in HOA Increased in HOA

(E)

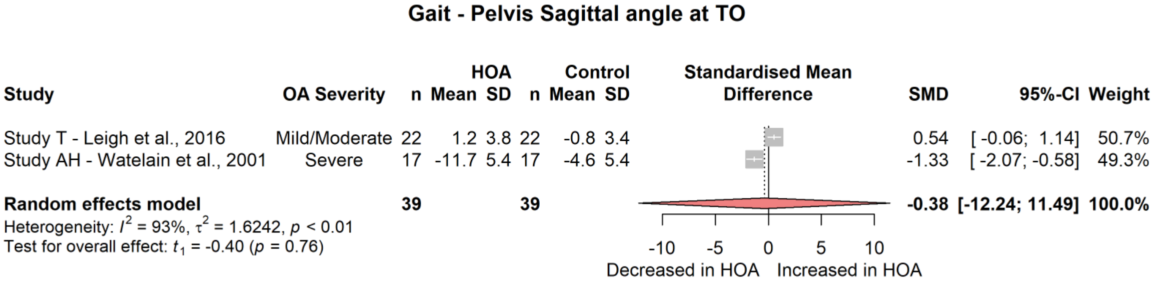
Gait - Pelvis Sagittal angle at TO
Study | OA Severity | HOA n Mean SD | Control n Mean SD | Standardised Mean Difference | SMD | 95%-CI | Weight
Study T - Leigh et al., 2016 Mild/Moderate 22 1.2 3.8 22 -0.8 3.4 0.54 [-0.06; 1.14] 50.7%
Study AH - Watelain et al., 2001 Severe 17 -11.7 5.4 17 -4.6 5.4 -1.33 [-2.07; -0.58] 49.3%
Random effects model 39 39 -0.38 [-12.24; 11.49] 100.0%
Heterogeneity: I^2 = 93%, τ^2 = 1.6242, p < 0.01
Test for overall effect: t_1 = -0.40 (p = 0.76)
-10 -5 0 5 10
Decreased in HOA Increased in HOA

Supplementary Figure 16. Forest plot of standardised and pooled effect sizes (random-effects-model) with I^2 heterogeneity statistics for (A) pelvis peak anterior tilt during gait cycle (GC), (B) pelvis peak anterior tilt during stance phase (ST), (C) pelvis peak posterior tilt during GC, (D) pelvis sagittal range of motion (ROM) across GC, (E) pelvis sagittal angle at toe-off (TO); during gait.

(A)

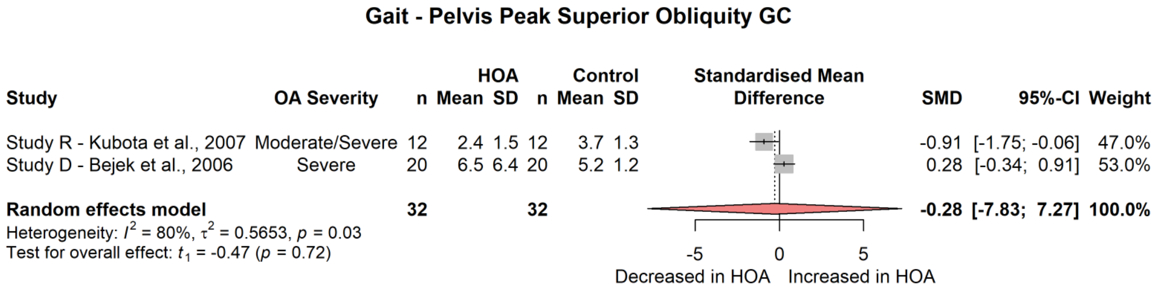

(B)

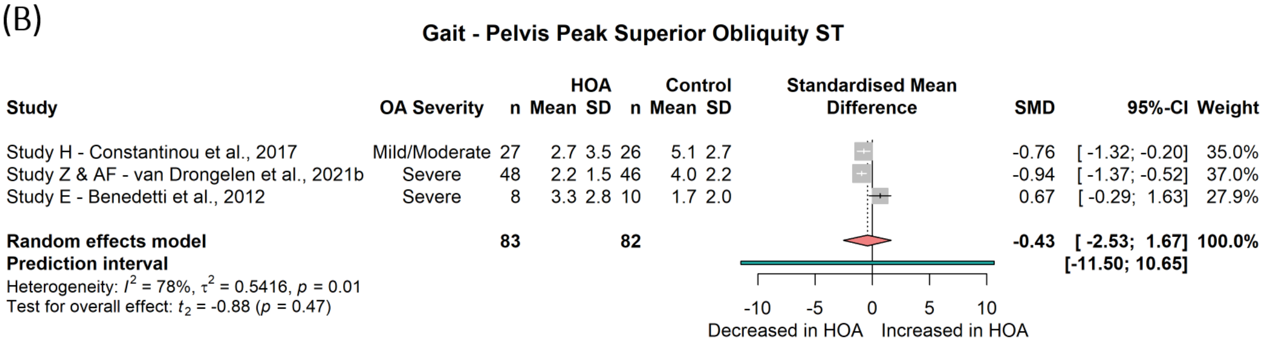

(C)

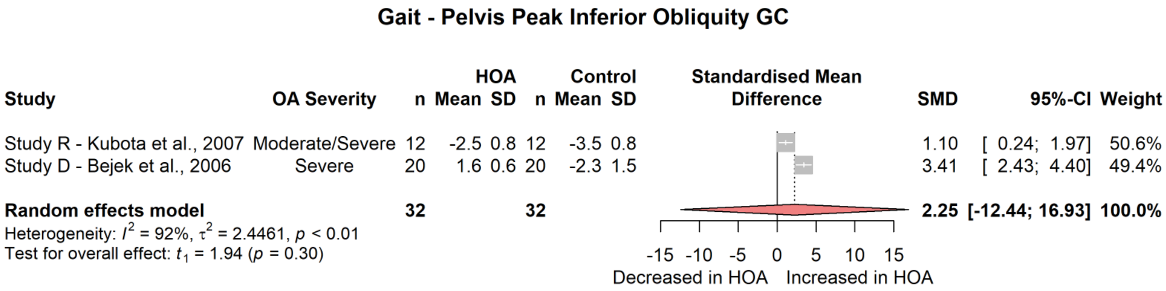

Supplementary Figure 17. Forest plot of standardised and pooled effect sizes (random-effects-model) with I² heterogeneity statistics for (A) pelvis peak superior obliquity during gait cycle (GC), (B) pelvis peak superior obliquity during stance phase (ST), (C) pelvis peak inferior obliquity during GC; during gait.

(A)

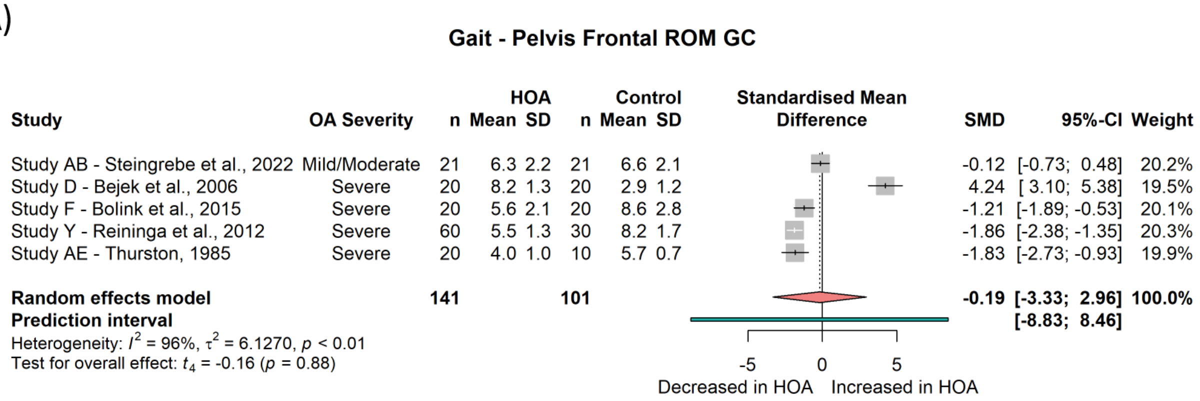

(B)

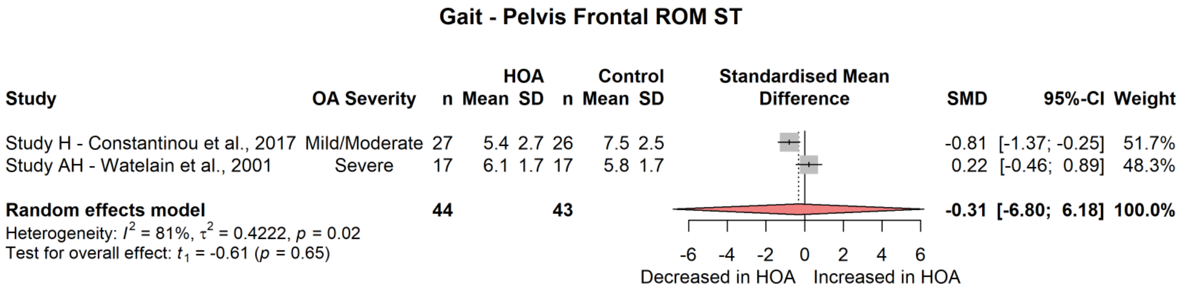

(C)

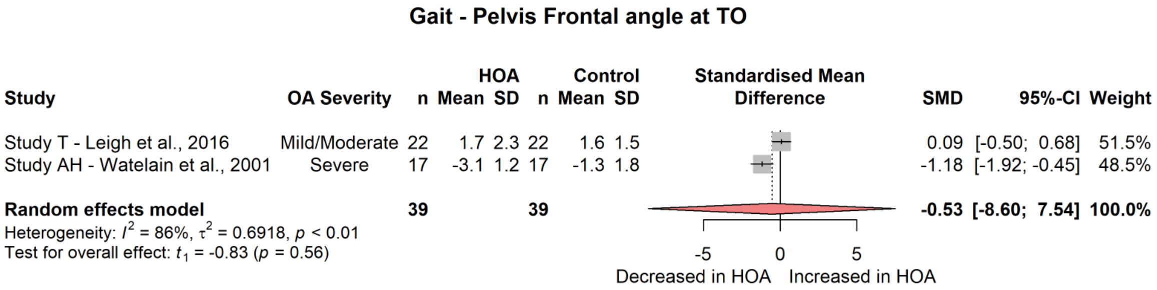

Supplementary Figure 18. Forest plot of standardised and pooled effect sizes (random-effects-model) with I² heterogeneity statistics for (A) pelvis frontal range of motion (ROM) across gait cycle (GC), (B) pelvis frontal ROM across stance phase (ST), (C) pelvis frontal angle at toe-off (TO); during gait.

(A)

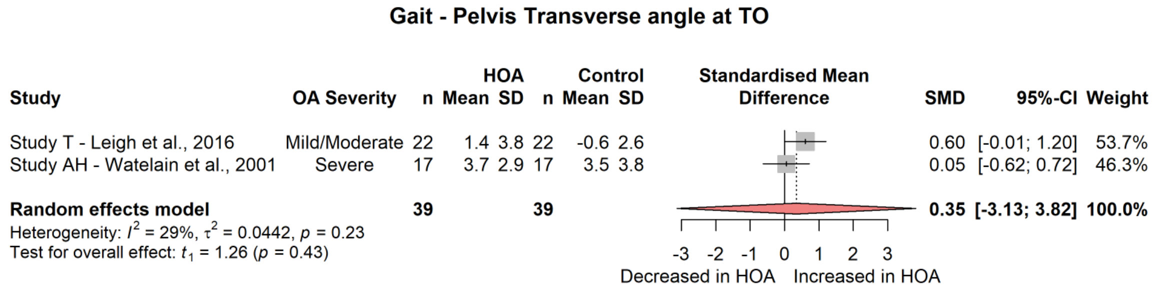

(B)

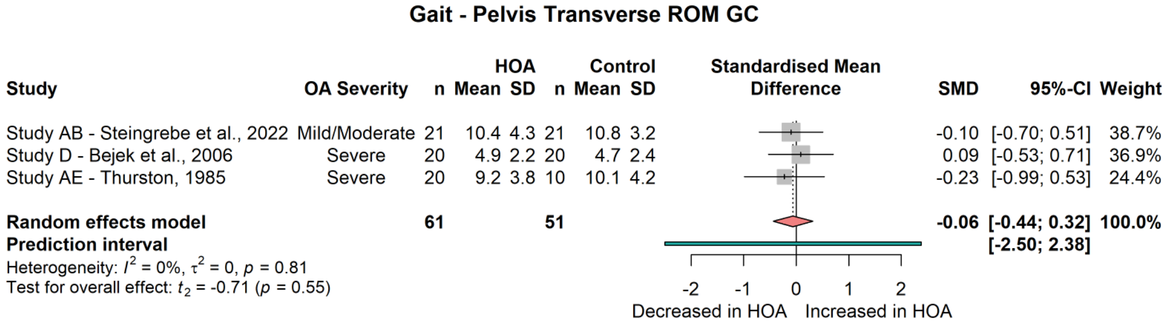

Supplementary Figure 19. Forest plot of standardised and pooled effect sizes (random-effects-model) with I² heterogeneity statistics for (A) pelvis transverse angle at toe-off (TO), (B) pelvis transverse range of motion (ROM) across gait cycle (GC); during gait.

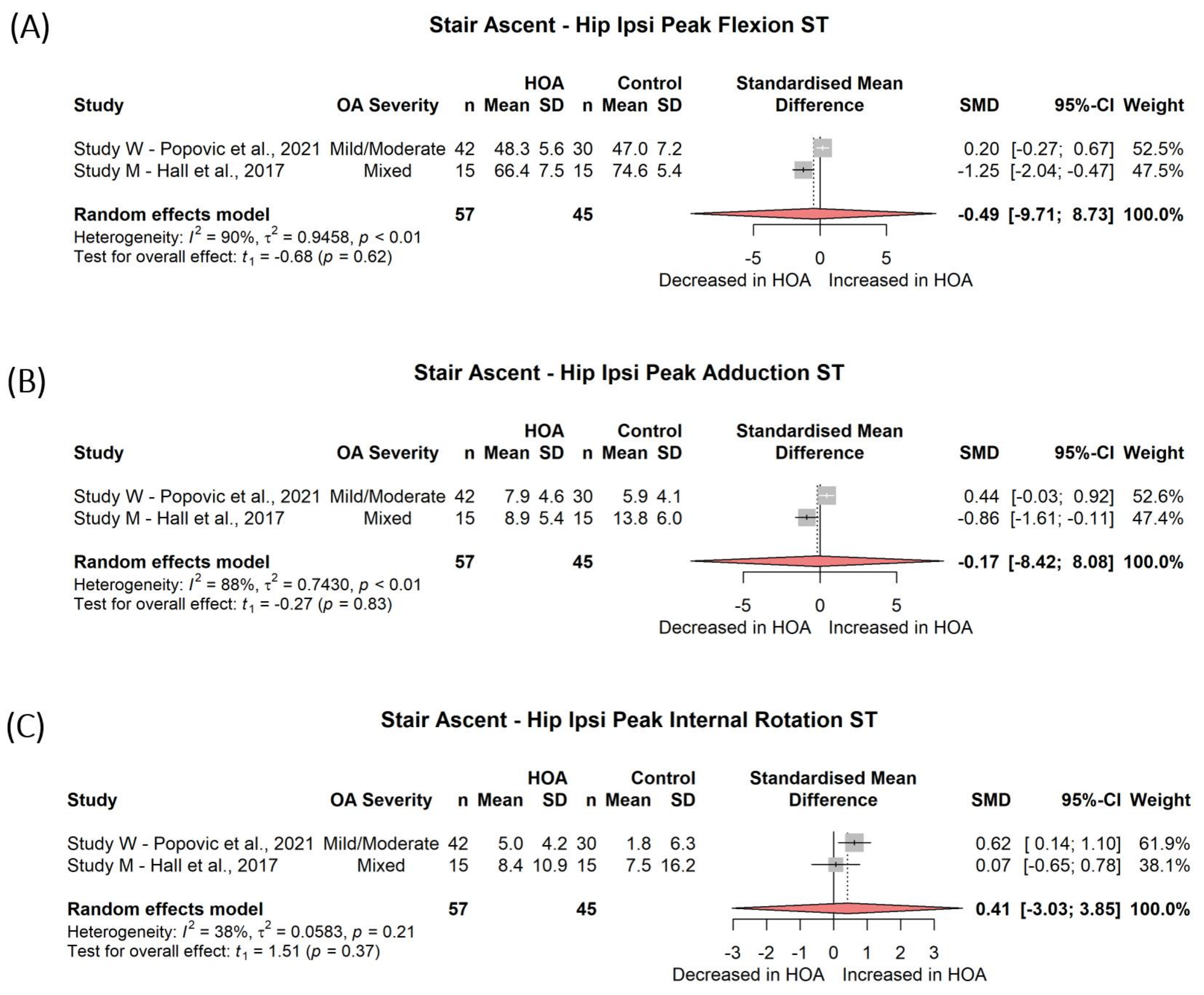

Supplementary Figure 20. Forest plot of standardised and pooled effect sizes (random-effects-model) with I² heterogeneity statistics for (A) ipsilateral peak hip flexion during stance phase (ST), (B) ipsilateral peak hip adduction during ST, (C) ipsilateral peak hip internal rotation during ST; during stair ascent.

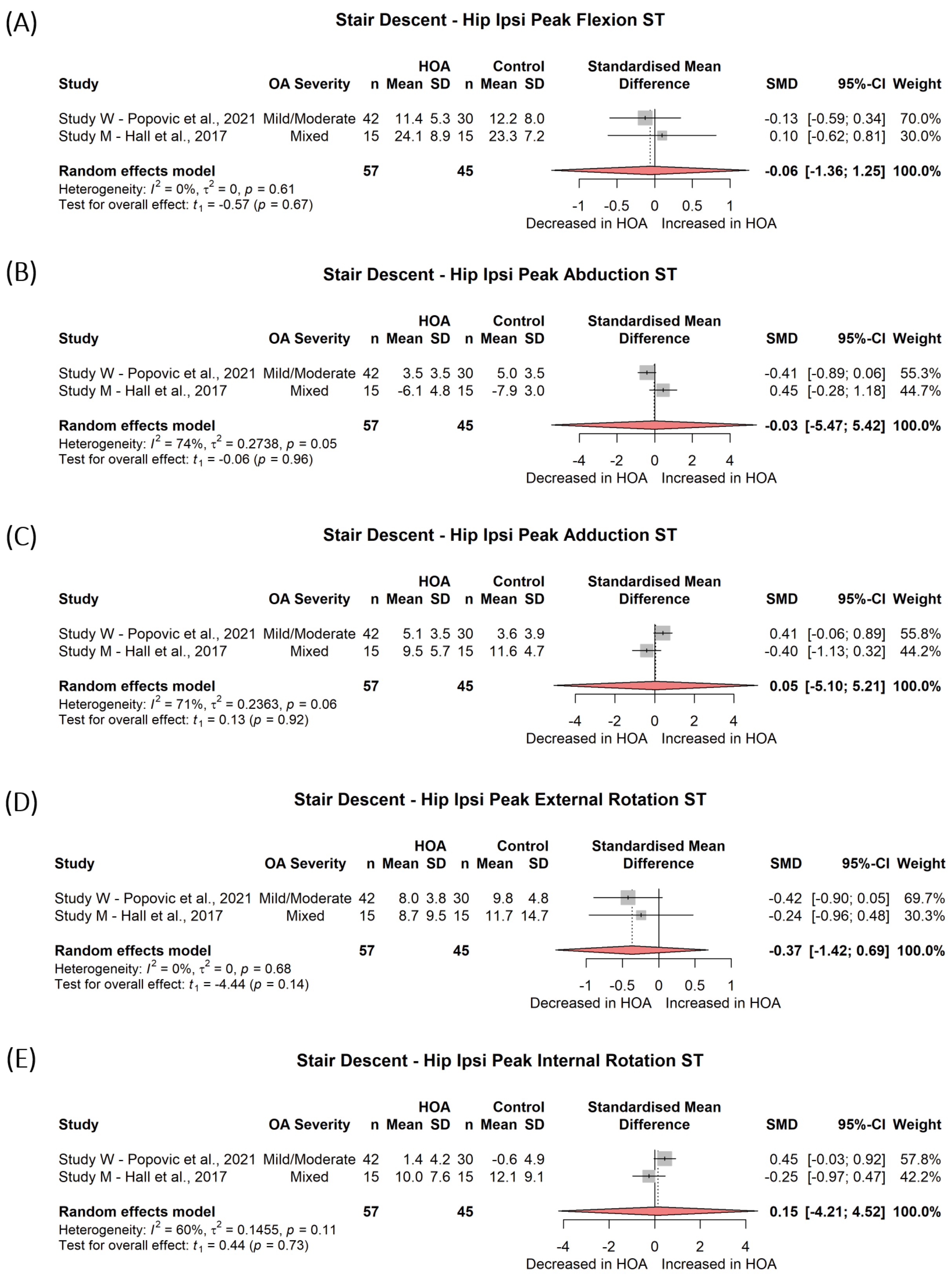

Supplementary Figure 21. Forest plot of standardised and pooled effect sizes (random-effects-model) with I^2 heterogeneity statistics for (A) ipsilateral peak hip flexion during stance phase (ST),

(B) ipsilateral peak hip abduction during ST, (C) ipsilateral peak hip adduction during ST, (D) ipsilateral peak hip external rotation during ST, (E) ipsilateral peak hip internal rotation during ST; during stair descent.
